# Lacking ASIC1a in ASIC4-positive amygdala/bed nucleus of the stria terminalis (BNST) neurons reduces anxiety and innate fear in mice

**DOI:** 10.1186/s12929-025-01138-6

**Published:** 2025-04-22

**Authors:** Ya-Chih Chien, Shing-Hong Lin, Cheng-Chang Lien, John N. Wood, Chih-Cheng Chen

**Affiliations:** 1 Program in Molecular Medicine, National Yang Ming Chiao Tung University and Academia Sinica, Taipei, Taiwan; 2 Institute of Biomedical Sciences, Academia Sinica, Taipei, Taiwan; 3 Institute of Neuroscience, National Yang Ming Chiao Tung University, Taipei, Taiwan; 4 The Wolfson Institute for Biomedical Research, University College London, WIBR UCL, Gower Street, London, WC1E 6BT UK; 5 Taiwan Mouse Clinic–National Comprehensive Phenotyping and Drug Testing Center, Academia Sinica, 128, Section , Academia Road, Taipei, 115 Taiwan; 6 Neuroscience Program of Academia Sinica, Academia Sinica, Taipei, Taiwan

**Keywords:** ASIC1a, ASIC4, Amygdala/BNST, Chemo-optogenetics, N-Glycosylation, Anxiety

## Abstract

**Background:**

Anxiety is an innate response in the face of danger. When anxiety is overwhelming or persistent, it could be considered an anxiety disorder. Recent studies have shown that acid-sensing ion channels (ASICs) represent a novel class of promising targets for developing effective therapies for anxiety. Especially, ASIC1a and ASIC4 of the ASIC family are widely expressed in the central nervous system and their gene knockouts result in reducing or enhancing anxiety-like responses in mice respectively. However, how ASIC1a and ASIC4 modulate anxiety-associated responses remains unknown.

**Methods:**

Here we combined chemo-optogenetic, conditional knockout, gene rescue, molecular biology and biochemistry, and electrophysiological approaches to probe the roles of ASIC4 and ASIC4-expressing cells in anxiety-associated responses in mouse models.

**Results:**

Chemo-optogenetically activating ASIC4-positive cells induced fear and anxiety-like responses in mice. Also, mice lacking ASIC4 (*Asic4*^*−/−*^) in the amygdala or the bed nucleus of the stria terminalis (BNST) exhibited anxiety-associated phenotypes. Conditional knockout of ASIC1a in ASIC4-positive cells reduced anxiety-associated behaviors. In situ hybridization analyses indicated that ASIC4 transcripts were highly co-localized with ASIC1a in the amygdala and BNST. We identified two glycosylation sites of ASIC4, Asn191 and Asn341, that were involved in interacting with ASIC1a and thus could modulate ASIC1a surface protein expression and channel activity. More importantly, viral vector-mediated gene transfer of wild-type ASIC4 but not Asn191 and Asn341 mutants in the amygdala or BNST rescued the anxiogenic phenotypes of *Asic4*^*−/−*^ mice.

**Conclusions:**

Together, these data suggest that ASIC4 plays an important role in fear and anxiety-related behaviors in mice by modulating ASIC1a activity in the amygdala and BNST.

## Background

Anxiety is a normal reaction when humans are faced with a threatening or stressful situation. When anxiety is overwhelming or persistent, it becomes an anxiety disorder. Accumulating evidence has revealed that the amygdala and associated neural circuits are an integrative center in regulating anxiety and fear responses [4, 9]. However, the molecular basis of the anxiety/fear circuitry remains largely unknown.

The amygdala complex is part of the neural circuitry that is critical for emotions [23]. Studies of animal models and patients with damage to the amygdala have shown its importance in emotional regulation. Many projection outputs from the amygdala have been found to mediate specific aspects of learning associated with positive and negative emotions. For instance, projection neurons of the basolateral amygdala that innervate into the periaqueductal grey [19], bed nucleus of the stria terminalis (BNST) [1], and ventral hippocampal nucleus [16], are responsible for generating anxiety-associated autonomic and motor responses.

Despite anxiety is a multifactorial disorder, acidosis due to CO_2_ inhalation is a well-known potent trigger for anxiety. Acid-sensing ion channels (ASICs) are a group of amiloride-sensitive proton-gated sodium channels widely expressed in the nervous system to sense extracellular acidification [10, 22, 35, 39]. The ASIC family contains at least four genes (Accn1-4) that encode six ASIC subtypes: ASIC1a, ASIC1b, ASIC2a, ASIC2b, ASIC3, and ASIC4 [30]. Among these subtypes, ASIC1a, ASIC1b, ASIC2a, and ASIC3 have differential sensitivity to protons, with an EC_50_ of 6.2–6.8, 5.1–6.2, 4.1–5.0, and 6.2–6.7 respectively, whereas ASIC2b and ASIC4 are not proton-sensitive [8, 21]. ASICs are trimeric channels consisting of 3 homomeric or heteromeric ASIC subtypes [24, 34].

In the brain, ASIC1a is the most dominant proton sensor involved in sensing acidosis due to stroke, seizure, synaptic transmission, and CO_2_ inhalation, etc. [12, 26, 38, 41]. A role for ASIC1a in regulating anxiety and fear responses has been highlighted because ASIC1a expression is particularly high in the amygdala and BNST [6, 37, 42]. CO_2_ inhalation lowers brain pH, which induces cerebral vasodilation and anxiety/fear responses by activating neuronal ASIC1a in the amygdala and BNST [15, 33]. ASIC1a is the principal ASIC subtype responding to acid signaling and regulating synaptic plasticity. In the amygdala, it plays a critical role in processing fear and anxiety-related stimuli, whereas in the BNST, it is pivotal in anxiety behaviors triggered by environmental factors such as CO_2_ exposure and acidosis [6, 33, 42]. The modulation of ASIC1a activity in these regions can significantly alter anxiety and fear responses, and it is a key target for therapeutic interventions in anxiety disorders. Understanding the role of ASIC1a in these neural circuits is vital for developing new treatments and interventions for anxiety-related conditions.

ASIC4 is another key player in regulating anxiety/fear responses [29]. Also known as spinal cord ASIC (SPASIC), ASIC4 is expressed throughout the central nervous system [2, 20]. Because protons cannot induce an inward current via ASIC4, we know little about its role. Previous studies have suggested a potential function of ASIC4 in modulating ASIC1a activity [11]. With Chinese hamster ovary cells used as a model, co-expression of ASIC4 and ASIC1a reduced the ASIC1a-mediated acid-induced inward current (*I*_ASIC1a_). ASIC4-knockout (ASIC4-KO) mice increased anxiety-like behaviors that contrasted with reduced anxiety-like response in ASIC1a-knockout mice [29]. However, as ASIC4 itself does not respond to acidosis, how ASIC4 modulates anxiety and fear responses remains unknown.

In the present study, we tested whether ASIC4 could modulate ASIC1a activity in the amygdala and BNST, crucial brain regions associated with anxiety and fear, thereby modulating anxiety and fear responses in mice. Combining chemo-optogenetic, conditional ASIC4-KO, electrophysiology, and adeno-associated virus (AAV)-driven gene rescue approaches, we revealed that ASIC1a channel activity in ASIC4-positive cells of the amygdala and BNST plays key roles in regulating anxiety and fear responses. ASIC4 acted to counterbalance ASIC1a activity. Consistent with this observation, ASIC4-KO mice exhibited increased anxiety levels and ASIC1a-KO mice decreased anxiety levels.

## Methods

### Animals

Wild-type C57BL/6JNarl mice were purchased from the National Laboratory Animal Center (NLAC, Taipei, Taiwan) and used as a backcross pool for genetically modified mutant mice. *Asic1a*^*floxed/floxed*^ (*Asic1a*^*f/f*^) mice were from Dr. Lien’s lab as described [40], *Asic1a*^*−/−*^ mice were generated by crossing protamine-Cre with *Asic1a*^*f/f*^ in our lab, and *Asic4*^*−/−*^ (homozygote *Asic4*^*CreERT2/CreERT2*^) mice were generated as described [29]. *Asic4*^*floxed/floxed*^ (*Asic4*^*f/f*^) mice were generated in John Wood’s lab. In brief, the exon 1 of *Asic4* was flanked by 2 loxP sites. Genotypes of offspring were determined from tail DNA by using PCR and the following primers: ASIC4-floxed-For: GAGCAGGATTGATAGGATAGC, ASIC4-floxed-rev: ATTTGCTACACTGTGTAGCTACAAG. *PCR program*: genomic DNA was amplified by first denaturing at 95 ˚C for 2 min followed by 35 cycles of denaturing at 95 ˚C for 30 s, annealing at 56 ˚C for 30 s, and extension at 72 ˚C for 1 min followed by a final extension at 72 ˚C for 10 min.

**Fig. 1:**
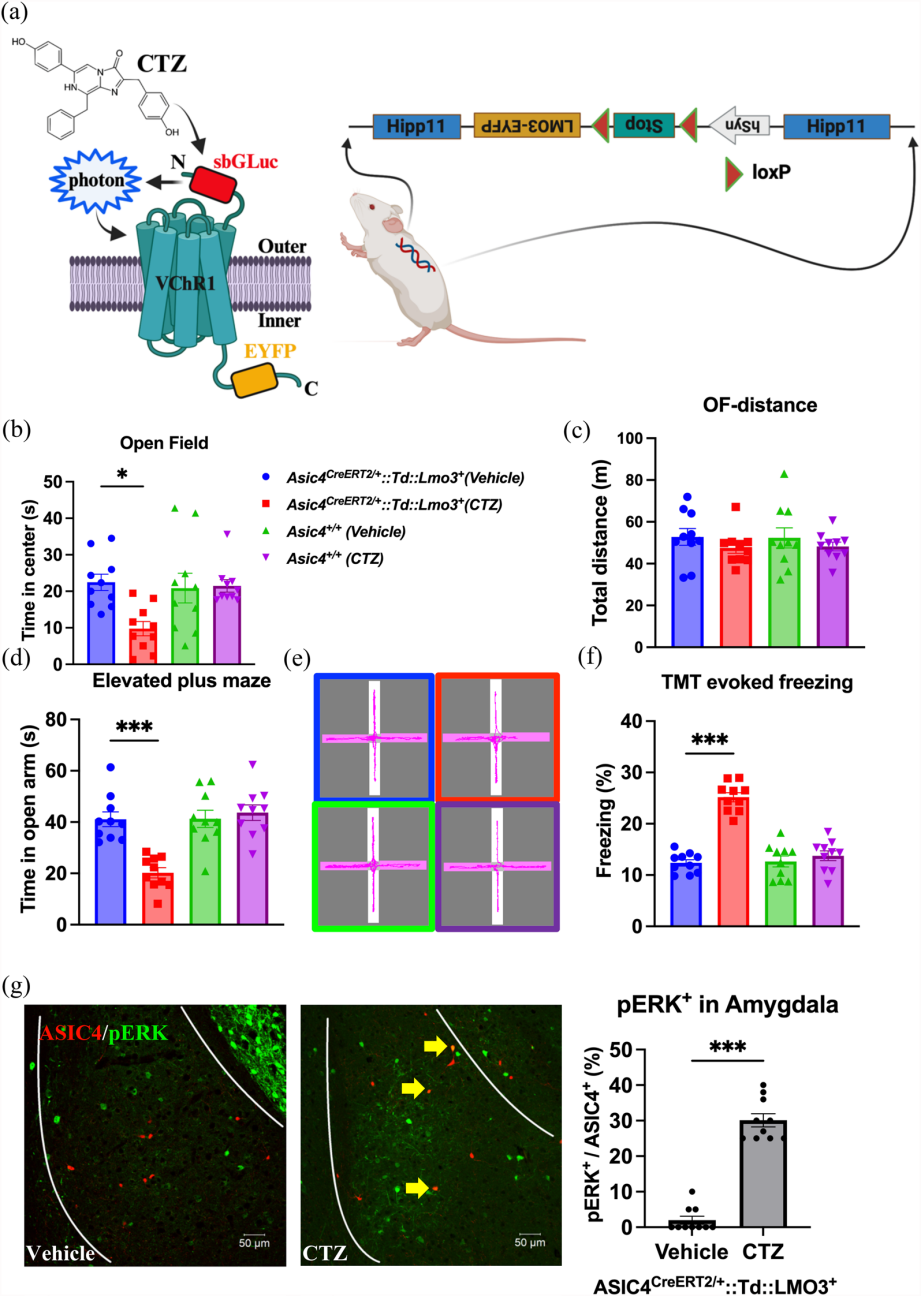
Chemo-optogenetic activation of ASIC4-positive cells induced anxiety-like behaviors in mice. **a** The design of Luminopsin 3 (LMO3), a fusing protein combining slow-burn luciferase (sbGLuc) and Volvox channelrhodopsin (VChR1), and genomic structure of the LMO3 reporter mouse. **b**, **c** Open-field test of effect of coelenterazine (CTZ) and vehicle treatment in *Asic4*^*CreERT2/*+^*::Td::Lmo3*^+^ mice (n = 10) and control *Asic4*^+*/*+^ mice (n = 10). **d** Elevated plus maze (EPM) test of effect of CTZ and vehicle treatment in mice (n = 10) and (**e**) image of maze. (f) 2,4,5-trimethylthiazoline (TMT) test of effect of CTZ and vehicle treatment in mice (n = 10). **g** Immunofluorescence staining of effect of CTZ and vehicle treatment on phosphorylated extracellular signal-regulated kinase (pERK) signal specific to ASIC4-positive cells in the amygdala of *Asic4*^*CreERT2/*+^*::Td::Lmo3*^+^ mice and quantification (n = 10). *p < 0.05, ***p < 0.001. Data are mean ± SEM

ROSA26R-lacZ mice (Jackson Lab: Stock no. 003309) and CAG-Td-tomato Cre reporter mice (Jackson Lab: Stock no. 007908) were crossed with *Asic4*^*CreERT2/*+^ transgenic mice. Luminopsin 3 (LMO3) reporter mice were generated in our lab. LMO3 is a fusing protein combining slow-burn luciferase and Volvox channelrhodopsin 1 (Fig. 1a). The construct of LMO3 was kindly gifted from Dr. Ute Hochgeschwender [3]. We modified the LMO3 gene under the human synapsin promoter (hSyn) with a STOP cassette flanked by loxP sites. The construct was insert between 3 K 5’ upstream and 1 K 3’ downstream of hipp11 loci, provided by Dr. Ching-Yen Tsai (Transgenic Core Facility, Institute of Molecular Biology, Academia Sinica, Taiwan) for homologous recombination. Genotypes of offspring were determined from tail DNA by using PCR and the following primers: LMO3-WT-forward: 5’-GATCAGGGCAGTCTGGTACTTC-3’, LMO3-WT-reverse: 5’-CCCACCAGCCTTGTCCTAATAAA-3’, LMO3-KI-reverse: 5’-GTTTGACACATCCTGCCCTTA-3’. *PCR program*: genomic DNA was amplified by first denaturing at 95 ˚C for 5 min followed by 36 cycles of denaturing at 95 ˚C for 30 s, annealing at 61 ˚C for 35 s, and extension at 72 ˚C for 40 s followed by a final extension at 72 ˚C for 5 min. Male mice at 14 to 18 weeks were recruited for behavioral tests from different litters of heterozygote inter-breeding. All mice were kept on a 12-h light–dark cycle, with all experiments performed during the light cycle.

### Tamoxifen induction and drug injection in mice

In experiments aimed at generating conditional knockouts of ASIC1a in ASIC4-expressing cells, mapping the expression of ASIC4, and manipulating the activity of ASIC4-positive cells, 3 Cre reporter mouse lines (*Asic1a*^*f/f*^, CAG-Td-tomato, LMO3) were each bred with *Asic4*^*CreERT2/*+^ mice. To restore ASIC4 expression, AAV-DIO-mCherry and/or AAV-DIO-ASIC4 were injected to specific brain regions of *Asic4*^*CreERT2/Cre−ERT2*^ mice. The resulting transgenic animals, aged 12 to 16 weeks, were treated with tamoxifen, administered by daily intraperitoneal injections of 2 mg for 7 consecutive days, and regular food chow was replaced with a special diet containing tamoxifen citrate (400 mg/kg) acquired from the Harlan laboratory. After behavioral testing, the animals were sacrificed, and tissue samples were collected for immunofluorescence staining and western blot analysis. Coelenterazine (CTZ) was from NanoLight Technology (CAT#303) (Pinetop, AZ, USA). For chemo-optogenetic study, CTZ (100 μg/100 μl) was intraperitoneally (i.p.) injected in mice 10 min before behavior testing.

### Assessment of anxiety-like behaviors and innate fear to a predator odor

Mice were habituated to the testing environment for 1 h before the behavioral test. The open field (OF) test was performed in a 48 × 48x48-cm, 4-wall chamber with 100-lx illumination. The mouse was released into the corner of the arena and allowed to freely explore for 20 min. Reduced time spent in the central area was considered an index of anxiety-like responses. We cleaned the instrument with 75% ethanol after each test to eliminate olfactory cues.

In the elevated-plus maze (EPM) test, an apparatus with 2 open arms (30 × 5 cm, with 1-cm ledges) and 2 closed arms (30 × 5 cm, with 15-cm walls) was used. The maze was elevated to a height of 50 cm above the floor during the task. Each animal was placed into the EPM facing a close arm and behaviors in the EPM were recorded for 5 min. Reduced time spent in the open arms was considered an index of anxiety-like responses. We cleaned the instrument with 75% ethanol after each test to eliminate olfactory cues. The time spent in open and closed arms, the time spent in the center area as well as distance travelled were automatically analyzed with use of TopScan (Clever Sys, Reston, VA, USA).

To measure freezing behavior, each mouse was placed in a near-infrared video conditioning chamber (Med Associates, St. Albans, VT, USA) and allowed to habituate for 3 min. In the predator-odor induced fear test, mice were placed in the chamber, and 20 μl 2,4,5-trimethylthiazoline (TMT, Phero Tech, Delta, BC, Canada), a synthetic analog of red fox feces, was applied onto a cotton ball and placed in a small beaker underneath the floor of the chamber [7]. Freezing response was defined as an absence of movement other than respiration and was recorded by using FreezeScan (CleverSys, Reston, VA, USA).

### Adeno-associated virus packing, stereotaxic microinjection

The Cre recombinase and enhanced yellow fluorescent protein (EYFP) was subcloned into the double-stranded AAV vector “pAAV-EMBL”. The production and quantification of AAV2/5, which contains the AAV5 capsids, AAV2 inverted terminal repeats** (**ITRs) and a CMV promoter driving Cre recombinase and EYFP, was performed in the AAV Core facility in the Institute of Biomedical Sciences, Academia Sinica, Taipei. The AAV stock was prepared in sterile PBS at 1.2 × 10^11^ vg/1 μl. For conditional knockout of ASIC4 in the different brain regions, double transgenic male mice with *Asic4*^*f/f*^ and at least a single allele of ROSAGt26-LacZ, received intracranial injection of AAV2/5-Cre or AV2/5-EFGP. For restoring study, the cDNA of mouse ASIC4 was derived from the plasmid pAAV-ASIC4-IRES-hrGFP and subcloned into the plasmid pAAV-hSyn-DIO-mCherry by replacing the mCherry. AAV-DIO-mCherry and AAV-DIO-ASIC4 were co-injected into specific brain areas. Before AAV injection, mice were anesthetized with 1.5% isoflurane and placed in the stereotaxic frame (Stoeling). Then, mice were inserted with a 30-G needle connected to a 10-μl Hamilton syringe into the target nucleus. An amount of 1 μl AAV was infused bilaterally at a rate of 0.2 μl/min with an infusion pump (DR instruments). The coordinates for AAV injection were amygdala = AP: − 1.2 mm; ML: ± 3.6 mm; DV: − 5.2 mm, bed nucleus of the stria terminalis, BNST = AP: 0.4 mm; ML: ± 1.0 mm; DV: − 4.3 mm, hippocampus = AP: − 2.0 mm; ML: ± 1.5 mm; DV: − 1.5 mm relative to bregma. After surgery, mice recovered for 2 weeks, then underwent tamoxifen-induced Cre recombination. To verify the size of the affected area and ensure the efficiency of restoration, after behavioral tests, mice were processed for x-gal staining, immunofluorescence staining or western blot analysis.

### Immunohistochemistry, x-gal staining and imaging

To analyze the tamoxifen-induced Cre recombination, *Asic4*^*CreERT2/*+^ heterozygotes were bred with CAG-Td-tomato reporter mice. Mice were perfused with 25 ml ice-cold tris buffer saline (TBS), followed by 25 ml of 4% paraformaldehyde (PFA) in TBS. Tissues were extracted and post-fixed in 4% PFA at 4 °C for 8 h. For X-gal staining, cryosections 16 μm thick were taken from mouse brains and mounted on gelatin-coated slides. Samples were soaked in the X-gal solution (0.15 M NaCl, 3.5 mM K_3_Fe(CN)_6_, 3.5 mM K_4_Fe(CN)_6_, 0.01 M PB, pH 7.4, 1 mM MgCl_2_, 0.3 M chloroquine, 0.01% sodium deoxycholate, 0.2% NP-40 and 1 mg/ml X-gal) at 37 °C for 6 h and counterstained with Neutral red. In the immunofluorescence procedure, after the perfusion protocol, tissues were immersed in 30% sucrose for cryoprotection at 4 °C for 24 h. They were then embedded in optimal cutting temperature (OCT) compound, rapidly frozen on dry ice, and immediately sectioned at 14 μm thick by use of a Leica CM3050S cryostat. The prepared slides were air-dried and preserved at − 80 °C for future analysis. The sections were washed 3 times with TBS + 0.1% Triton X-100 (TBST) and blocked for 1 h at room temperature in TBST containing 3% bovine serum albumin and 5% normal serum of the second antibody host. To investigate coelenterazine (CTZ)-induced extracellular signal-regulated kinase (ERK) phosphorylation (pERK) expression, mice were first anesthetized, then underwent intraperitoneal (i.p.) injection of CTZ. Mice were subjected to the perfusion protocol at 3 min after CTZ injection, and brain samples were collected for immunofluorescence staining. Primary antibodies were diluted in the blocking solution, which was incubated overnight at 4 °C. Subsequently, sections were washed 3 times with PBST and incubated for 1 h at room temperature with secondary antibodies (diluted 1:400). The primary antibody and its titers were as follows: rabbit-anti-PERK (diluted 1:300), mouse-anti-calretinin (Swant, 1:300), rabbit-anti-vasoactive intestinal peptide (Immunostar, 1:500). All sections were imaged under a confocal microscope (Zeiss 780).

### In situ Hybridization

To obtain brain tissues, mice were first anesthetized by intraperitoneal injection of 1.3 mg/kg of urethane (Sigma-Aldrich, St. Louis, MO, USA). Then, they were subjected to perfusion with 25 ml ice-cold PBS, followed by 25 ml of 4% PFA in PBS. In our in situ hybridization procedure, we used brain cryosections that were 12 μm thick, which were affixed to VWR Microslides (VWR, Radnor, PA, USA). We followed the manufacturer's guidelines for the RNAscope fluorescent multiplex reagent kit, which was supplied by Advanced Cell Diagnostics (Newark, CA, USA). The RNA probes used for in situ hybridization were specifically designed and provided by Advanced Cell Diagnostics. For duplex hybridization, the probes were for ASIC1a (Cat. 462,381-C2) targeting region 178–749 and ASIC4 (Cat. 511,971-C1) targeting on region 2–890.

### Chinese hamster ovary (CHO) cell culture, transfection and constructs

CHO-K1 cells were purchased from ATCC, and cells between passages 3 and 15 were used. CHO cells were grown in Ham’s F-12 nutrient mix medium supplemented with 10% fetal bovine serum in a humidified 5% CO_2_ incubator. For transfection experiments, CHO cells were seeded onto 35-mm dishes at 5*10^5^ cells/cc. One day after seeding, plasmid was transfected by using Lipofectamine 2000 as per the manufacturer’s instruction. Briefly, cells were rinsed with PBS three times to remove the culture medium, the transfection medium (Opti-MEM) for 1 h, and cells were incubated with the freshly prepared lipofectamine-plasmid mixture for 24 h. The cDNA of mouse ASIC1a was subcloned into the end of the plasmid pCMV-mCherry-P2A to generate pCMV-mCherry-P2A-ASIC1a as previously described [27]. The pAAV-ASIC4-IRES-hrGFP plasmid was used as a template to generate ASIC4 mutants (ASIC4_N191A_,_N243A_,_N341A_,_N376A_, ASIC4_N191A_, ASIC4_N243A_, ASIC4_N341A_, ASIC4_N376A_) via site-directed mutagenesis with TransStart FastPfu Fly DNA Polymerase (TransGen Biotech Co., Beijing). All constructs were verified by sequencing.

### Whole-cell patch clamp recording

Whole-cell patch-clamp recording was performed as described [28] to test the acid-induced inward current in the mCherry- and GFP-positive CHO cells transfected with ASIC1a and ASIC4. The recording cells were kept in artificial cerebrospinal fluid ACSF containing (in mM) 130 NaCl, 5 KCl, 1 MgCl_2_, 2 CaCl_2_, 10 Glucose, and 20 HEPES, adjusted to pH 7.4 with NaOH; osmolality 298–310 mOsm. The pH 5.0 acidic ACSF was titrated with 2-[N-morpholino]ethanesulfonic acid, and pH 5.6, pH 6.2, pH and 6.8 acidic ACSF was titrated with NaOH. Acidic or neutral ACSF was applied via a glass pipette 50 μm from the cell and with gravity controlled by a VC-6 six-channel value (Warner Instrument). The recording electrode was filled with internal solution containing (in mM) 100 KCl, 2 Na_2_-ATP, 0.3 Na_3_-GTP, 10 EGTA, 5 MgCl_2_, and 40 HEPES, adjusted to pH 7.4 with KOH; osmolality 298–310 mOsm. The pipette resistance was 6 to 8 MΩ. ASIC1a channels were triggered by a drop in pH from 7.4 to given values every 3 min to allow for complete recovery of the channel from desensitization. Whole-cell currents were elicited by a drop in pH from 7.4 to different pHs at a holding potential of − 70 mV. For pH activation curves, the ACSF flowing out of one barrel of the perfusion system was pH 7.4, and the ACSF flowing out of the second barrel was switched to pH 6.8, 6.2, 5.6 and 5.0 sequentially. Acid-triggered currents at each pH were normalized to the peak current activated at pH 5.0. Normalized values were fitted to the Hill equation by using Prism 9 software to obtain EC_50_ values and Hill coefficients. To determine the time constant of the desensitizing portion of the ASIC currents, pH 5.0-activated currents were fitted by a single, standard exponential equation using Clamp- fit 10.2. We used a CED1401 MK2 converter (Cambridge Electronic Design, Cambridge, UK) to perform the recording. The series resistance was compensated 70% in voltage-clamping recording with Axopatch 700B compensation circuitry. For each recording, the transfected cell would receive a 5-s acidic ACSF challenge for 3 times, with an intersection of 25 s. All recordings were performed at room temperature (22–25 ˚C).

### Plasma membrane preparation and western blot analysis

Cell-surface proteins of transfected CHO cells were extracted by using the Hook Cell Surface Protein Isolation kit (G-Biosciences, St. Louis, MO, USA). Briefly, cell-surface proteins were labeled with Hook-sulfo-NHS-SS-Biotin. In this approach, a biotin-tag/streptavidin affinity column was used to extract only proteins exposed on the outer plasma membranes of intact cells and were analyzed by western blot analysis. Because of a small amount of protein samples, we pooled samples from 3 dishes of 1 × 10^6^ cells. Tissue punches for western blot analysis were taken bilaterally from the amygdala, BNST and hippocampus. For each tissue, a total 30-μg lysate was run on a Bis–Tris gel and transferred to a PVDF membrane for western blot analyses. Primary antibodies used were goat polyclonal anti-ASIC1 antibody (Santa Cruz Biotechnology 1:1000), goat polyclonal anti-ASIC4 antibody (Santa Cruz Biotechnology 1:1000), rabbit polyclonal anti-beta Tubulin antibody (Millipore 1:10,000), mouse monoclonal anti-sodium potassium ATPase (Abcam 1:1000), mouse monoclonal anti-Actin antibody (Millipore 1:10,000). Secondary antibodies used were rabbit anti-goat IgG (GeneTex 1:5000), goat anti-rabbit IgG (GeneTex 1:5000) and goat anti-mouse IgG (GeneTex 1:5000). The blots were imaged by using the VisionWorks Life Science Software.

### Amygdala cell preparation

Following AAV injection and tamoxifen induction, *Asic4*^*−/−*^ mice were sacrificed and their brains were rapidly isolated for sectioning. Mouse brain was sectioned into slices with 2 mm thick via a steel brain matrix (Stoelting-51386, Illinois, USA). The amygdala was isolated from sections 6–8 via a polished 18G needle puncture. The amygdala tissues were then digested in 1 mL 1 × HBSS containing 0.25 mM papain (Sigma-P4762) at 37 °C for 30 min. Following digestion, the dissociated cells were washed once with 1X HBSS and subsequently used for plasma membrane preparation and Western blot analysis.

### Data analysis and statistics

Data in all figures are presented as mean ± SEM. Two-tailed Student *t* test, one-way or two-way ANOVA followed by a post-hoc Holm-Sidak test was used to determine statistical differences in data from behavior, electrophysiology, and immunostaining assays. p < 0.05 was considered statistically significant. Statistical comparisons involved using Prism 10.

## Results

### Chemo-optogenetic activation of ASIC4-expressing cells induced anxiety-like behaviors

Previously, we demonstrated that *Asic4*^*−/−*^ mice showed increased innate fear responses and anxiety-related behaviors as compared with wild-type mice [29]. To understand whether activating ASIC4-expressing cells could modulate fear and anxiety responses, we applied chemo-optogenetic approaches by using *Asic4*^*CreERT2*^*::LMO3* mice (Fig. 1a). After tamoxifen induction, *Asic4*^*CreERT2*^*::LMO3* mice were injected with CTZ or vehicle, left idle for 10 min, then underwent behavioral tests to measure fear responses. In open-field tests, CTZ injection significantly reduced time spent in the center zone in *Asic4*^*CreERT2*^*::LMO3* but not wild-type *Asic4*^+*/*+^ mice as compared with vehicle treatment (two-way ANOVA, treatment: F_(1,36)_ = 5.147, p = 0.03; genotype: F_(1,36)_ = 3.618, p = 0.07; interaction: F_(1,36)_ = 6.275, p = 0.02) (Fig. 1b). In contrast, CTZ did not have any effect on locomotion activity in both genotypes (Fig. 1c). In elevated-plus maze (EPM) tests, CTZ injection significantly reduced the time spent in the open arm in *Asic4*^*CreERT2*^*::LMO3* but not wild-type mice as compared with vehicle treatment (two-way ANOVA, treatment: F_(1,36)_ = 12.26, p = 0.001; genotype: F_(1,36)_ = 13.6, p < 0.001; interaction: F_(1,36)_ = 18.67, p < 0.001) (Fig. 1d, e). In TMT-evoked freezing tests, CTZ injection significantly increased freezing responses in *Asic4*^*CreERT2*^*::LMO3* but not wild-type mice as compared with vehicle treatment (two-way ANOVA analysis, treatment: F_(1,36)_ = 60.66, p < 0.001; genotype: F_(1,36)_ = 38.96, p < 0.001; interaction F_(1,36)_ = 42.84, p < 0.001) (Fig. 1f). Next, we demonstrated whether CTZ could specifically activate ASIC4-positive cells by examining ERK phosphorylation as a surrogate marker. In *Asic4*^*CreERT2*^*::LMO3* mice, 3 min after CTZ injection, pERK-immunoreactive cells were observed in the amygdala and co-localized with td-tomato signals (e.g., ASIC4-positive cells) (Fig. 1g). Together, activation of ASIC4-expressing cells induced anxiogenic behavioral responses in mice.

### Conditional knockout of ASIC4 in amygdala/BNST affected anxiety/fear responses in mice.

**Fig. 2:**
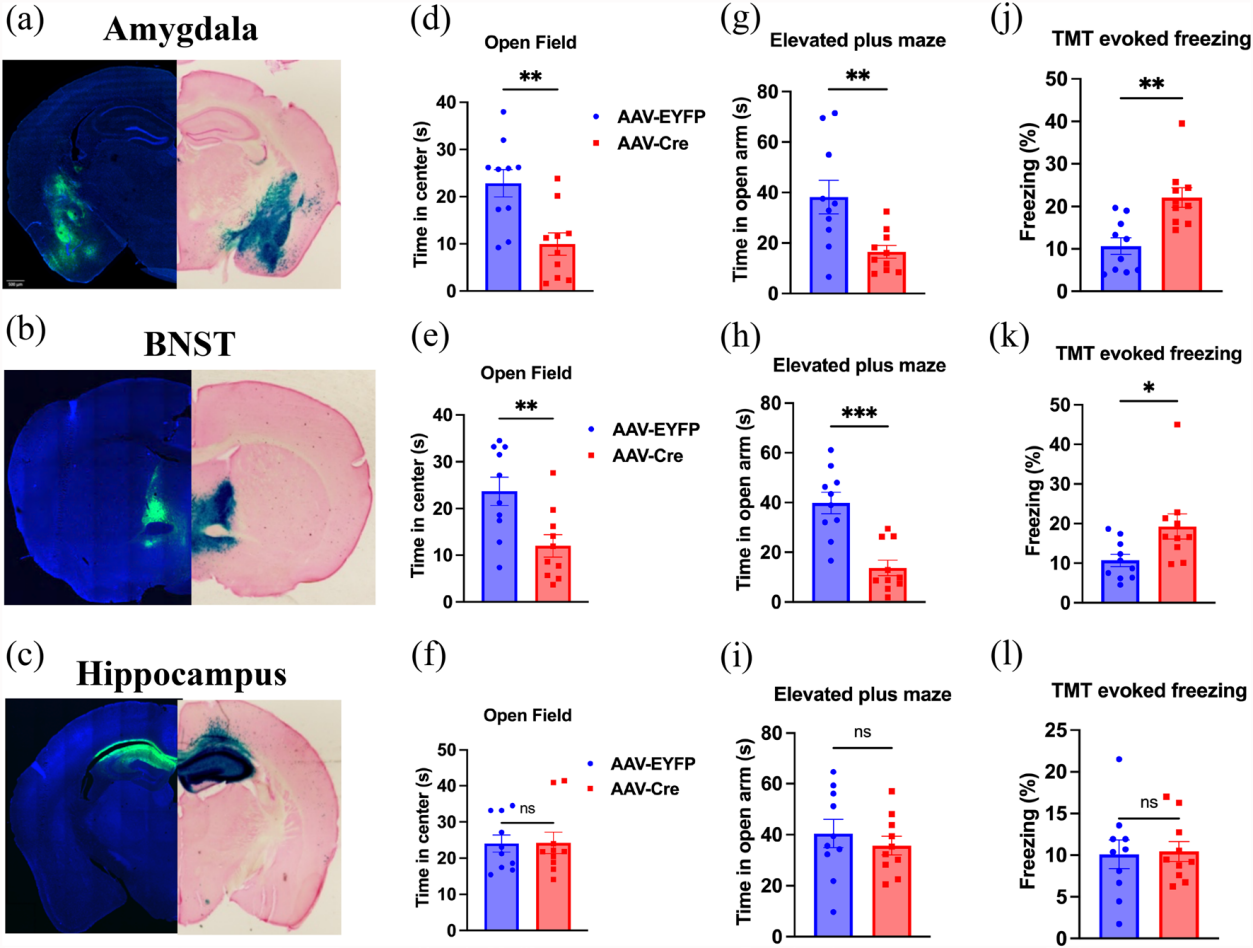
Conditional ASIC4 knockout in the amygdala and BNST increase anxiety-like responses. **a**–**c** Representative brain images of adeno-associated virus (AAV)-mediated gene expression in ROSA26R-lacZ mice. Mice injected with AAV-EYFP or AAV-Cre were visualized with green fluorescence (left side) and lacZ staining (right side) in the amygdala (n = 10), bed nucleus of the stria terminalis (BNST) (n = 10), and hippocampus (n = 10). **d**–**f** Open-field test. **g**–**i** EPM test. **j–l** TMT test (n = 10). *p < 0.05, **p < 0.01, ***p < 0.001; ns, not significant. Data are mean ± SEM

Because ASIC4 is widely expressed in the central nervous system [29], we next probed which brain regions were critical for ASIC4’s role in modulating anxiety and fear responses. We used *Asic4*^*f/f*^*::*ROSA26R-lacZ mice and bilaterally injected an AAV containing the coding sequence of enhanced yellow fluorescent protein (EYFP, AAV-EYFP) or Cre recombinase (AAV-Cre) into the amygdala, BNST, or hippocampus to locally delete ASIC4 gene. As expected, intense X-gal and EYFP signals were detected in all targeted brain regions (Fig. 2a–c). Mice with brain region-specific ASIC4 KO underwent anxiety-related behavior tests. In open field tests, mice with ASIC4 KO (AAV-Cre) in the amygdala or BNST spent less time in the center zone as compared with mice that received control AAV-EYFP (p < 0.01, amygdala; p < 0.01, BNST) (Fig. 2d–f). This behavioral change was not found in mice with ASIC4 KO in the hippocampus (p = 0.960). Next, in the EPM tests, mice injected with AAV-Cre in the amygdala or BNST spent less time in the open arm as compared with mice injected with AAV-EYFP (p < 0.01, amygdala; p < 0.001, BNST), with no difference when AAV-Cre was injected in the hippocampus (p = 0.820) (Fig. 2g–i). Last, in TMT-evoked freezing tests, mice injected with AAV-Cre in the amygdala or BNST with AAV-Cre showed significantly increased freezing as compared with mice injected with AAV-EYFP (p < 0.01, amygdala; p < 0.05, BNST), with no difference when AAV-Cre was injected in the hippocampus (p = 0.868) (Fig. 2j–l). Together, deletion of ASIC4 in the amygdala and BNST led to an anxiogenic phenotype in mice.

### Co-localization of ASIC4 and ASIC1a in the amygdala and BNST of mice.

**Fig. 3:**
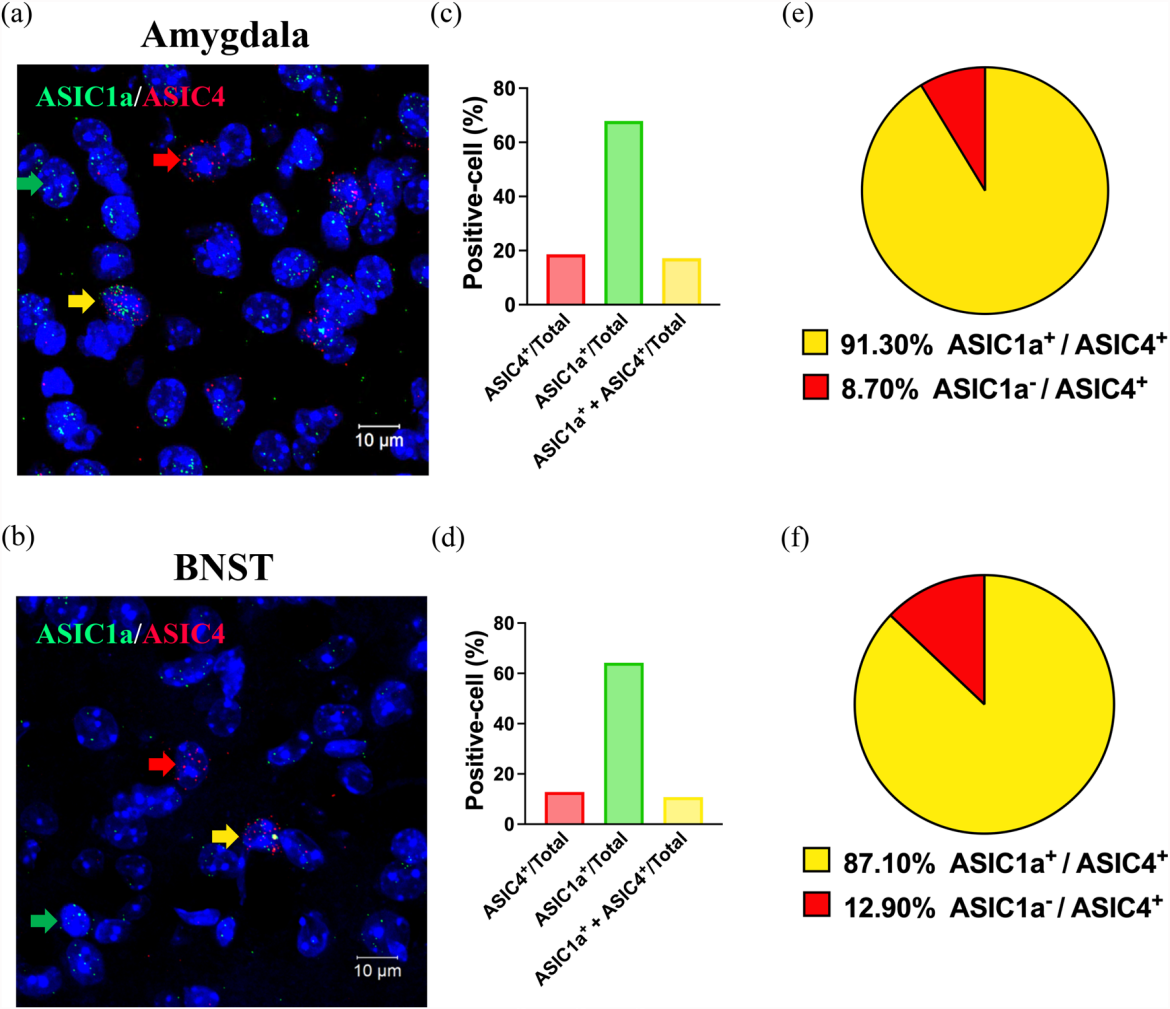
Co-localization of ASIC4 and ASIC1a in the amygdala and BNST of mice. Representative image by RNAscope of co-expression of *Asic4* and *Asic1a* in the (**a**) amygdala and (**b**) BNST. Red arrows indicate *Asic4* + cells, green yellow arrows indicate *Asic1a* + cells, and yellow arrows indicate cells co-expressing *Asic4* and *Asic1a* mRNA transcripts. **c** Quantification analysis showed *Asic4* and *Asic1a* expression in the amygdala was 19% (46/255) and 68% (173/255) of total cells, respectively. Co-expression of *Asic4* and *Asic1a* was 16% (42/255) of total cells. **d** Quantification analysis showed *Asic4* and *Asic1a* expression in the BNST was 13% (31/234) and 64% (150/234) of total cells, respectively. Co-expression of *Asic4* and *Asic1a*. was 12% (27/234) of total cells. **e**, **f** Pie chart of *Asic4*-expressing cells that were *Asic1a*-positive in the amygdala (**e**), and BNST (**f**) respectively. Three independent trials of the experiment were performed in 3 mice

Previous studies have shown that ASIC4 can regulate ASIC1a surface expression in an heterologous expression system [11], so we wondered whether ASIC4 could modulate ASIC1a activity and thus influence anxiety-like response. Therefore, we used RNAscope to determine whether ASIC4 is co-expressed with ASIC1a in the amygdala and BNST. We detected both ASIC1a and ASIC4 transcripts in the amygdala (Fig. 3a). ASIC1a and ASIC4 were expressed in 68% (173/255) and 19% (46/255) of total cells in the amygdala (Fig. 3c). Also, ASIC4 transcripts highly co-localized with ASIC1a (Fig. 3e). We detected both ASIC1a and ASIC4 transcripts in the BNST (Fig. 3b). ASIC1a and ASIC4 were expressed in 64% (150/234) and 13% (31/234) of total cells in the BNST (Fig. 3d). As in the amygdala, in the BNST, ASIC4 transcripts highly co-localized with ASIC1a (Fig. 3f). These results suggest that ASIC4 could interact with ASIC1a and thus modulate ASIC1a activity in vivo.

### Conditional ***Asic1a*** knockout in ***ASIC4***-positive cells effectively elicits ***Asic1a*** knockout (***Asic1a***^−/−^) anxiolytic phenotypes.

**Fig. 4:**
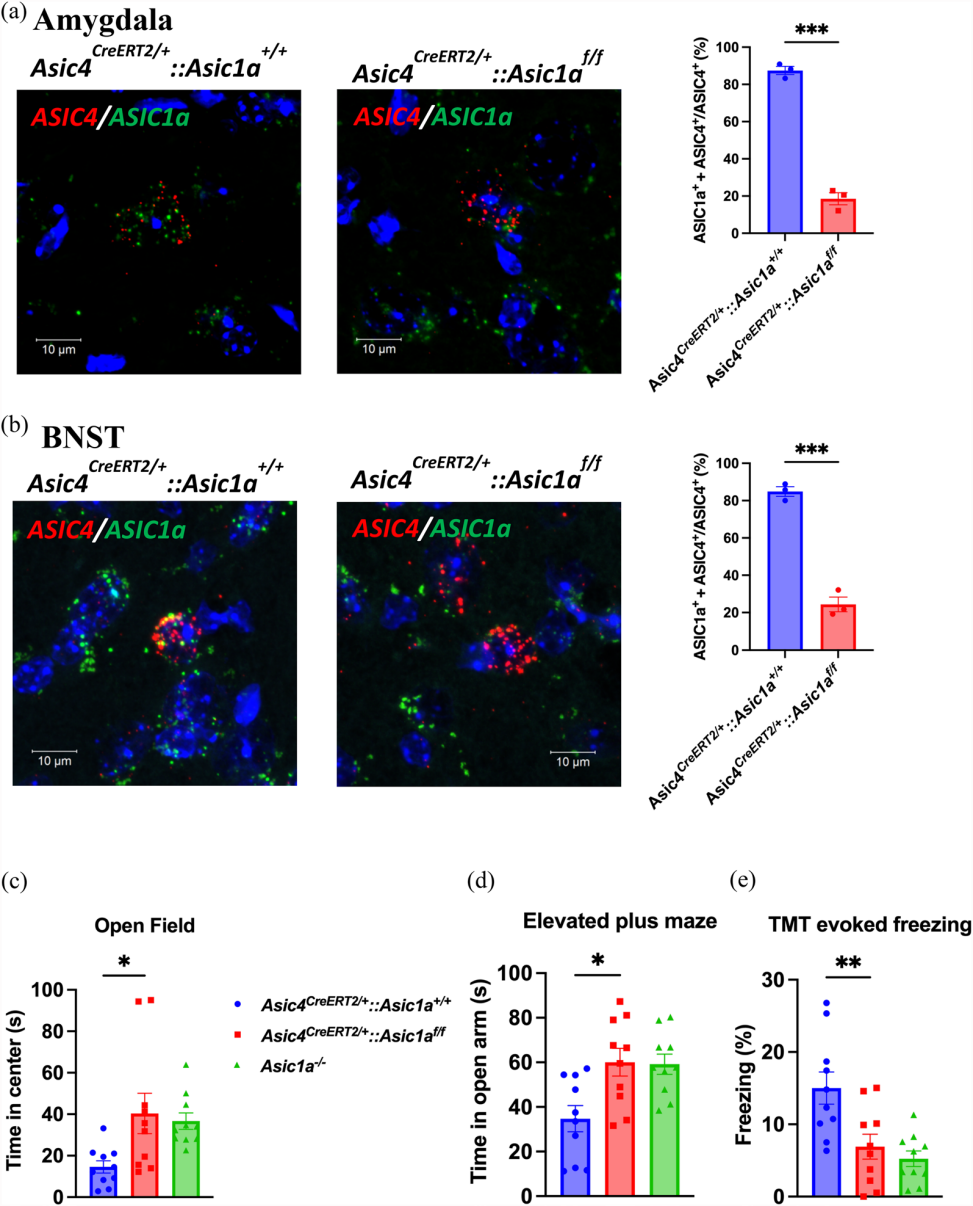
Conditional *Asic1a* knockout in *ASIC4*-positive cells effectively elicits *Asic1a* knockout (*Asic1a*^−/−^) anxiolytic phenotypes. **a** Representative images by RNAscope and quantitative analysis of co-expression of *Asic4* and *Asic1a* in the Amygdala of *Asic4*^*CreERT2/*+^::*Asic1a*^+*/*+^ and *Asic4*^*CreERT2/*+^::*Asic1a*^*f/f*^ mice (n = 3). **b** Representative images by RNAscope and quantitative analysis of co-expression of Asic4 and Asic1a in the BNST of *Asic4*^*CreERT2/*+^::*Asic1a*^+*/*+^ and *Asic4*^*CreERT2/*+^::*Asic1a*^*f/f*^ mice (n = 3). **c** Open-field test of anxiety responses after tamoxifen induction in *Asic4*^*CreERT2/*+^::*Asic1a*^*f/f*^, *Asic1a*^*−/−*^ and *Asic4*^*CreERT2/*+^::*Asic1a*^+*/*+ *mice*^ (n = 10 each). **d** EPM test. **e** TMT test. *p < 0.05, **p < 0.01, ***p < 0.001 *Asic4*^*CreERT2/*+^::*Asic1a*^+*/*+^ vs. *Asic4*^*CreERT2/*+^::*Asic1a*^*f/f*^*.* Data are mean ± SEM

To investigate whether ASIC4 could modulate fear responses via ASIC1a expression, we used *Asic4*^*CreERT2/*+^*::Asic1a*^*Floxed/Floxed*^ transgenic mice to conditionally knockout ASIC1a in ASIC4-expressing cells by tamoxifen induction for 7 days. After tamoxifen induction, the co-localizations of *Asic1a* and *Asic4* transcripts were significantly reduced in the amygdala and BNST of *Asic4*^*CreERT2/*+^*::Asic1a*^*Floxed/Floxed*^ mice as compared with those in *Asic4*^*CreERT2/*+^*::Asic1a*^+*/*+^ mice (Fig. 4a, b). In open field tests, time spent in the center zone was greater for both *Asic1a*^*−/−*^ and *Asic4*^*CreERT2/*+^*::Asic1a*^*Floxed/Floxed*^ mice than *Asic4*^*CreERT2/*+^*::Asic1a*^+*/*+^ mice (one-way ANOVA analysis: F_(2,27)_ = 4.866, p = 0.02, Fig. 4c). In the EPM tests, time spent in the open arm was greater for both *Asic1a*^*−/−*^ and *Asic4*^*CreERT2/*+^*::Asic1a*^*Floxed/Floxed*^ mice than wild-type mice (one-way ANOVA analysis: F_(2,27)_ = 6.631, p = 0.005, Fig. 4d). In TMT evoked freezing tests, freezing response was lower for both *Asic1a*^*−/−*^ and *Asic4*^*CreERT2/*+^*::Asic1a*^*Floxed/Floxed*^ mice than wild-type mice (one-way ANOVA analysis: F_(2,27)_ = 9.067, p < 0.001, Fig. 4e). Hence, conditional knockout *Asic1a* in ASIC4-expressing cells could fully replicate the anxiety phenotypes of *Asic1a*^*−/−*^ mice.

### Effect of ASIC4 mutations on ASIC1a-mediated current

**Fig. 5:**
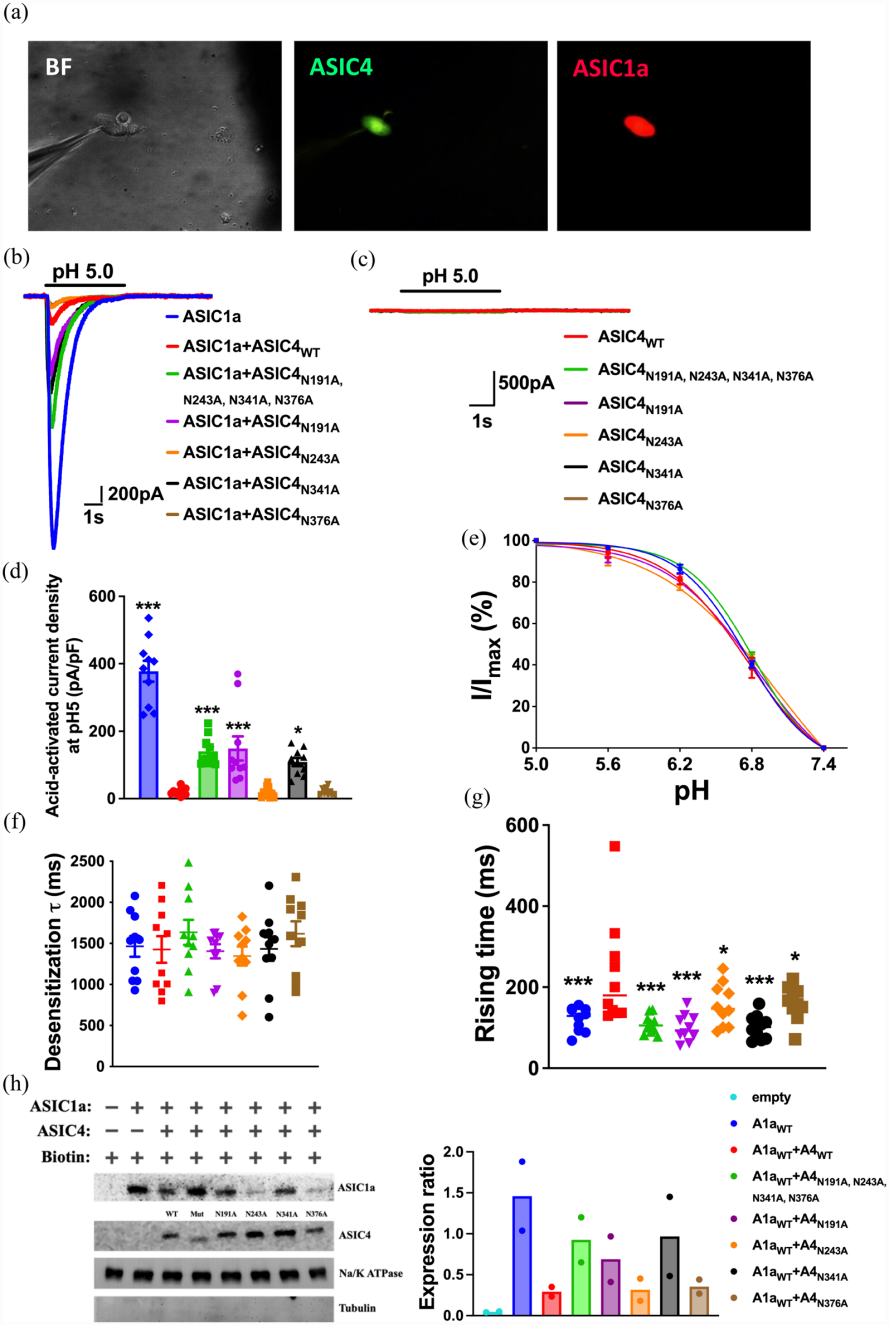
Effect of ASIC4 mutations on ASIC1a channel activity. Co‐expression of ASIC4 and ASIC1a reduced acid-induced currents. **a** A representative image of Chinese hamster ovary (CHO) cells co-transfected with both pCMV-mCherry-P2A-ASIC1a and pCMV-ASIC4-IRES-hrGFP. **b** Representative current traces of acid (pH 5.0)-induced currents in CHO cells transfected with ASIC1a, ASIC1a + ASIC4_WT_, ASIC1a + ASIC4_N191A, N243A, N341A, N376A_, ASIC1a + ASIC4_N191A_, ASIC1a + ASIC4_N243A_, ASIC1a + ASIC4_N341A_ or ASIC1a + ASIC4_N376A_ (n = 10 cells each). Co-expression of ASIC1a with ASIC4_WT_ or its mutants reduced ASIC1a-mediated acid-induced current (*I*_ASIC1a_). **c** Acid (pH 5.0) did not induce a current in CHO cells transfected with ASIC4_WT_, ASIC4_N191A, N243A, N341A, N376A_, ASIC4_N191A_, ASIC4_N243A_, ASIC4_N341A_, or ASIC4_N376A_ (n = 5 cells each). **d** Quantification of acid-induced peak current density in transfected CHO cells. **e** pH dependence of the *I*_ASIC1a_ in transfected CHO cells. **f** Desensitization time constant (τ) of* I*_ASIC1a_ at pH 5.0 in transfected CHO cells. **g** Activation time constant (τ) of* I*_ASIC1a_ at pH 5.0 in transfected CHO cells. **h** Western blot analysis of surface ASIC1a expression in transfected groups (n = 2). *p < 0.05, ***p < 0.001 vs. ASIC1a + ASIC4_WT_. Data are mean ± SEM

We next probed how ASIC4 could regulate ASIC1a surface expression and channel activity. Previous studies have shown that N-glycosylation of ASIC1a is involved in stabilizing channel expression and trafficking [25]. According to sequence analysis, the extracellular domain of ASIC4 contains more N-glycosylation sites than other ASIC subtypes. Therefore, we hypothesized that N-glycosylation of ASIC4 might play important roles in regulating ASIC1a function. We used CHO cells as a heterologous expression system to test how ASIC4 and its N-glycosylation mutants (N191A, N243A, N341A, N376A) could affect ASIC1a channel activity (Fig. 5a). As expected, whole-cell patch clamp recordings revealed acid-induced currents in CHO cells transfected with ASIC1a plasmids but not cells transfected with wild-type ASIC4 (ASIC4_WT_) or its N-glycosylation mutants (Fig. 5b, c). Therefore, we tested how these ASIC4 N-glycosylation mutants would affect the ASIC1a-mediated inward current (*I*_ASIC1a_) induced by pH 5.0 buffer (blue trace) in CHO cells. Co-transfection of ASIC1a and ASIC4_WT_ reduced the *I*_ASIC1a_ (red trace), ASIC1a co-transfected with N-glycosylation mutants had differential effects on *I*_ASIC1a_ amplitude (Fig. 5b). The effect of ASIC4_WT_ on lowered *I*_ASIC1a_ current density was significantly recovered on co-transfection of ASIC1a with ASIC4_N191A, N243A, N341A, N376A_, ASIC4_N191A_, or ASIC4_N341A_ mutants (one-way ANOVA analysis: F_(6,69)_ = 5.2, p < 0.001) but not ASIC4_N243A_ or ASIC4_N376A_ mutants (Fig. 5d). However, co-transfection of ASIC4_WT_ or its mutants did not alter the *I*_ASIC1a_ activation curve in EC_50_ (F_(4,45)_ = 2.054, p = 0.10) or slope (F_(4,45)_ = 1.945, p = 0.12) (Fig. 5e). Further analyses revealed that the desensitization time constants of the *I*_ASIC1a_ (in pH 5.0) were not altered with co-transfection of ASIC4_WT_ or its mutants (Fig. 5f). The time constants of the* I*_ASIC1a_ were only increased on co-transfection of ASIC4_WT_ but not all mutants (Fig. 5g). To further probe the protein expression contributing to the *I*_ASIC1a_ amplitude, we treated CHO cells with biotinylation and extracted the plasma membrane fraction to analyze the surface expression of ASIC1a and ASIC4 on western blot analysis (Fig. 5h). As expected, the surface expression of ASIC1a in CHO cells was significantly reduced when ASIC1a was co-transfected with ASIC4_WT_. In contrast, co-transfection of the ASIC4_N191A, N243A, N341A, N376A_ mutant did not alter the ASIC1a surface expression. In comparison, co-transfection of ASIC1a with single site mutants of ASIC4_N243A_ or ASIC4_N376A_ reduced ASIC1a surface expression as compared with co-transfection with ASIC4_N191A_ or ASIC4_N341A_, which suggests that ASIC1a surface expression is highly associated with *I*_ASIC1a_ amplitude.

### Restoring ASIC4 expression in amygdala neurons rescues deficits in anxiety-like response in ASIC4-KO mice.

**Fig. 6:**
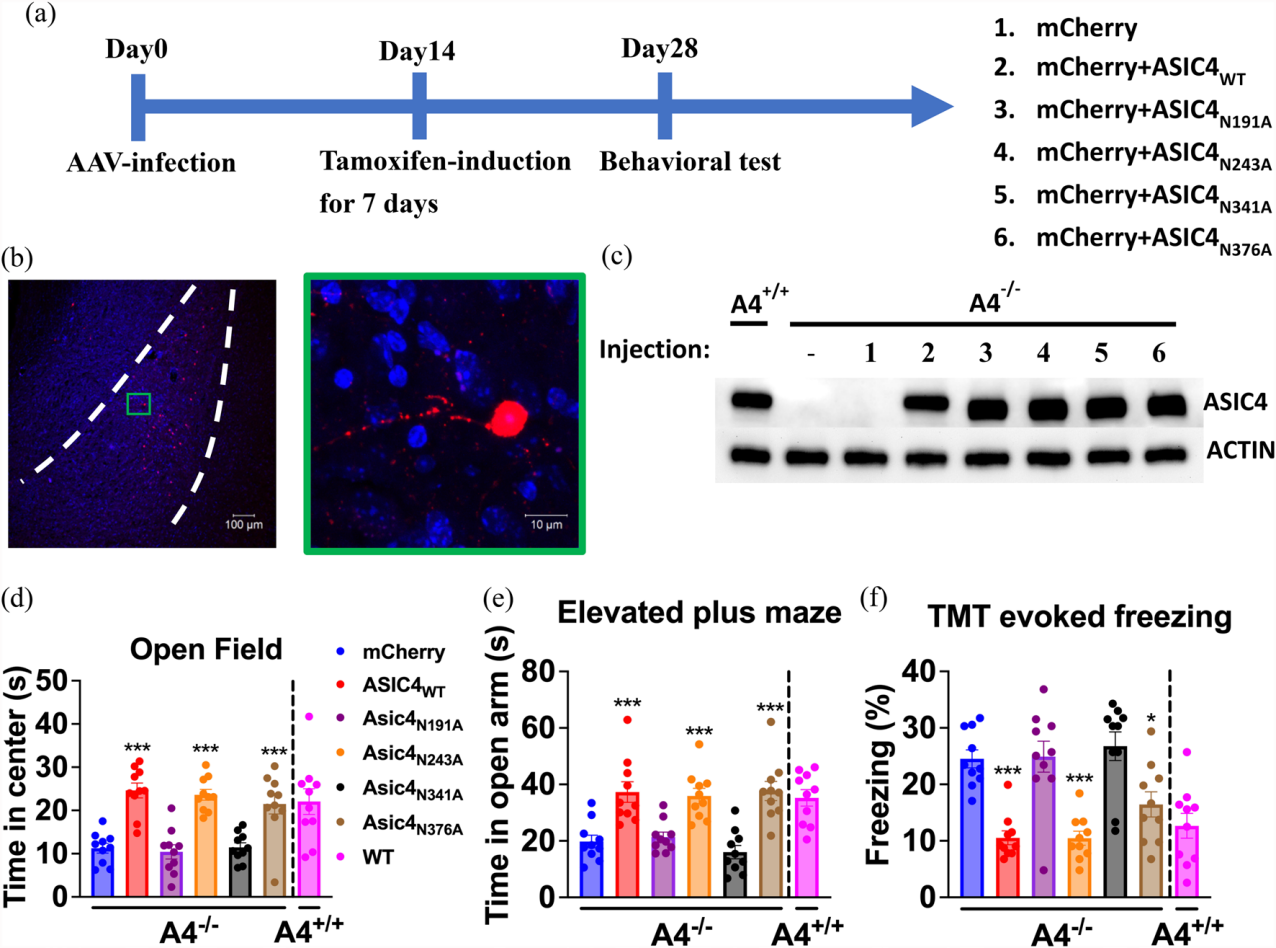
Restoring ASIC4 expression in the amygdala rescued anxiety phenotypes of *Asic4* knockout (*Asic4*^*−/−*^) mice. **a** The experimental flowchart for restoring ASIC4 expression in specific brain regions. **b** Representative image of *Asic4*^*−/−*^ amygdala injected with AAV-DIO-mCherry. **c** Western blot analysis of ASIC4 protein in the amygdala of *Asic4*^+*/*+^ mice and *Asic4*^*−/−*^ mice transfected with AAV-DIO-ASIC4_WT(or Mutants)_; actin was a loading control. **d** Effect of restoring amygdala ASIC4 (or its mutants) on *Asic4*^*−/−*^ mouse behaviors in open-field test, (**e**) EPM test, and (**f**) TMT-evoked freezing test (N = 10 mice in each group). ***p < 0.001 vs. mCherry. Data are mean ± SEM

We tested whether restoration of ASIC4 expression in related brain regions of ASIC4-KO mice could rescue their anxiety/fear phenotypes (Fig. 6a). We restored ASIC4 expression in putative ASIC4-expressing neurons in the amygdala by bilaterally injecting AAV containing the reverse coding sequence of ASIC4 (AAV-DIO-ASIC4) and/or mCherry (AAV-DIO-mCherry) driven by a neuron-specific synapsin promoter in *Asic4*^*CreERT2/CreERT2*^ (*Asic4*^*−/−*^) mice. After tamoxifen induction, ASIC4-expressing cells were visualized with mCherry signals in the amygdala (Fig. 6b). The AAV-driven expression of ASIC4_WT_ or its mutants was Cre-dependently expressed in ASIC4-expressing neurons and validated by western blot analysis (Fig. 6c). In open-field tests, center entry time significantly differed among *Asic4*^*−/−*^ mice transfected with ASIC4_WT_ or its mutants in bilateral amygdala (one-way ANOVA: F_(5.54)_ = 18.36, p < 0.001, Fig. 6d). On post-hoc analysis, the anxiety-like phenotype was significantly rescued on amygdala transfection with ASIC4_WT_, ASIC4_N243A_, or ASIC4_N376A_ but not ASIC4_N191A_ or ASIC4_N341A_ versus mice transfected with mCherry. Similarly, in EPM tests, open-arm entry time significantly differed among groups (one-way ANOVA: F_(5,54)_ = 13.05, p < 0.001), and the anxiety-like phenotype was significantly rescued on amygdala transfection with ASIC4_WT_, ASIC4_N243A_, or ASIC4_N376A_ but not ASIC4_N191A_ or ASIC4_N341A_ (Fig. 6e). In TMT-evoked freezing tests, freezing time significantly differed among groups (one-way ANOVA: F_(5,54)_ = 13.65, p < 0.001), and fear responses were significantly rescued on amygdala transfection with ASIC4_WT_, ASIC4_N243A_, or ASIC4_N376A_ but not ASIC4_N191A_ or ASIC4_N341A_ (Fig. 6f).

### Restoring ASIC4 expression in BNST neurons rescues deficits in anxiety-like response in ASIC4-KO mice.

**Fig. 7:**
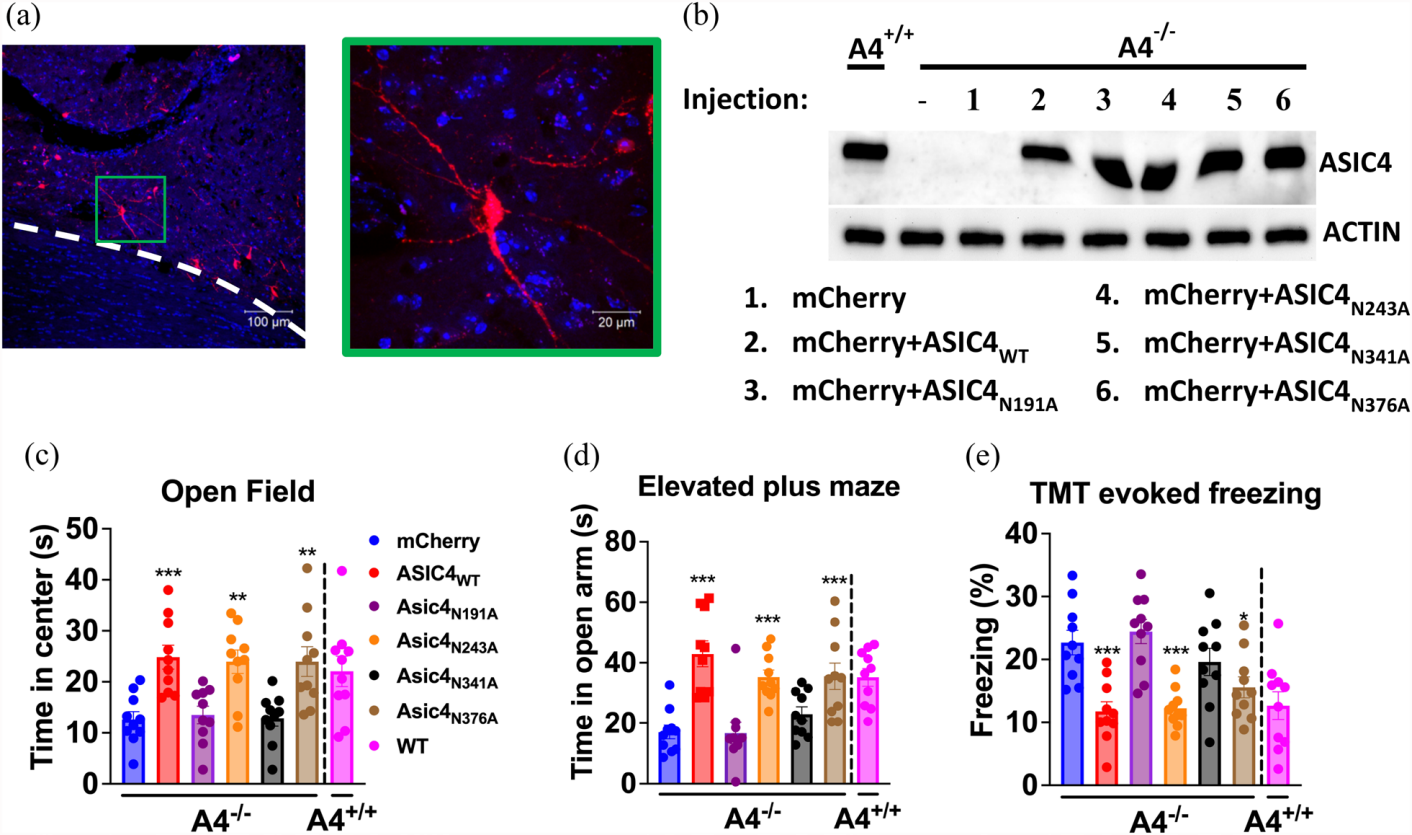
Restoring ASIC4 expression in the BNST rescued anxiety phenotypes of *Asic4* knockout (*Asic4*^*−/−*^) mice. **a** Representative image of *Asic4*^*−/−*^ BNST injected with AAV-DIO-mCherry. **b** Western blot analysis of ASIC4 protein in the BNST of *Asic4*^+*/*+^ mice and *Asic4*^*−/−*^ mice transfected with AAV-DIO-ASIC4_WT(or Mutants)_; actin was a loading control. **c** Effect of restoring BNST ASIC4 (or its mutants) on *Asic4*^*−/−*^ mouse behaviors in open-field test, (**d**) EPM test, and (**e**) TMT-evoked freezing test (N = 10 mice in each group). **p < 0.01, *** p < 0.001 vs. mCherry. Data are mean ± SEM

We next tested whether restored ASIC4 expression in the BNST could also rescue the anxiety/fear phenotypes of *Asic4*^*−/−*^ mice. We restored ASIC4 expression in putative ASIC4-expressing cells in the bilateral BNST by injecting AAV-DIO-ASIC4 and/or AAV-DIO-mCherry in *Asic4*^*CreERT2/Cre−ERT2*^ (*Asic4*^*−/−*^) mice (Fig. 7a) and validated its expression by western blot analysis (Fig. 7b). In open-field tests, center entry time significantly differed among *Asic4*^*−/−*^ mice transfected with ASIC4_WT_ or its mutants in the bilateral BNST (one-way ANOVA: F_(5.54)_ = 8.525, p < 0.001, Fig. 7c). On post-hoc analysis, the anxiety-like phenotype was significantly rescued on BNST transfection with ASIC4_WT_, ASIC4_N243A_, or ASIC4_N376A_ but not ASIC4_N191A_ or ASIC4_N341A_ versus mice transfected with mCherry. In EPM tests, open-arm entry time significantly differed among groups (one-way ANOVA: F_(5,54)_ = 10.99, p < 0.001), and the anxiety-like phenotype was significantly rescued on BNST transfection of ASIC4_WT_, ASIC4_N243A_, or ASIC4_N376A_ but not ASIC4_N191A_ or ASIC4_N341A_ (Fig. 7d). In TMT-evoked freezing tests, freezing time significantly differed among groups (one-way ANOVA: F_(5,54)_ = 9.456, p < 0.001), and fear responses were significantly rescued on BNST transfection of ASIC4_WT_, ASIC4_N243A_, or ASIC4_N376A_ but not ASIC4_N191A_ or ASIC4_N341A_ (Fig. 7e).

### Restoring ASIC4 expression in hippocampus neurons has no effect on anxiety-like response in ASIC4-KO mice.

**Fig. 8:**
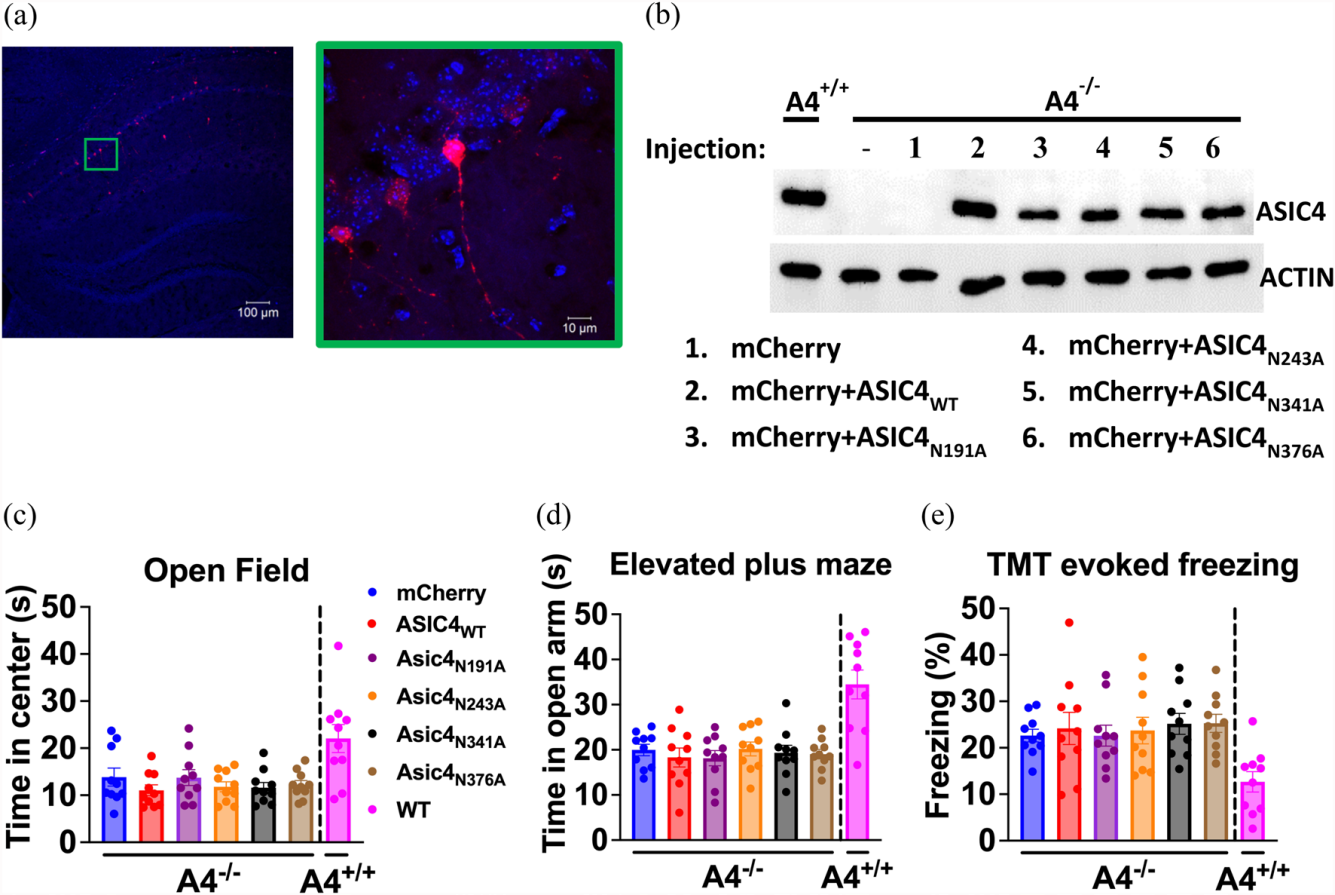
Restoring ASIC4 in the hippocampus showed no effect anxiety and fear in *Asic4* knockout (*Asic4*^*−/−*^). **a** Representative image of *Asic4*^*−/−*^ BNST injected with AAV-DIO-mCherry. **b** Western blot analysis of ASIC4 protein in the BNST of *Asic4*^+*/*+^ mice and *Asic4*^*−/−*^ mice transfected with AAV-DIO-ASIC4_WT(or Mutants)_; actin was a loading control. **c** Effect of restoring hippocampus ASIC4 (or its mutants) on *Asic4*^*−/−*^ mouse behaviors in open-field test, (**d**) EPM test, and **e** TMT-evoked freezing test (N = 10 in each group). **p < 0.01, ***p < 0.001 vs. mCherry. Data are mean ± SEM

To validate the brain region-specific effect of ASIC4 restoration, we restored ASIC4 expression in putative ASIC4-expressing cells in the bilateral hippocampus by injecting AAV-DIO-ASIC4 and/or AAV-DIO-mCherry in *Asic4*^*CreERT2/Cre−ERT2*^ (*Asic4*^*−/−*^) mice (Fig. 8a) and validated their expression by western blot analysis (Fig. 8b). In open-field tests, center entry time did not significantly differ among groups (one-way ANOVA: F_(5.54)_ = 0.7416, p = 0.6, Fig. 8c). In EPM tests, open arm entry time did not significantly differ among groups (one-way ANOVA: F_(5.54)_ = 0.2872, p = 0.92, Fig. 8d). In TMT-evoked fear tests, freezing time did not significantly differ among groups (one-way ANOVA: F_(5.54)_ = 0.2276, p = 0.95, Fig. 8e).

### The expression of neuronal markers in ASIC4-positive cells in the amygdala and BNST.

**Fig. 9:**
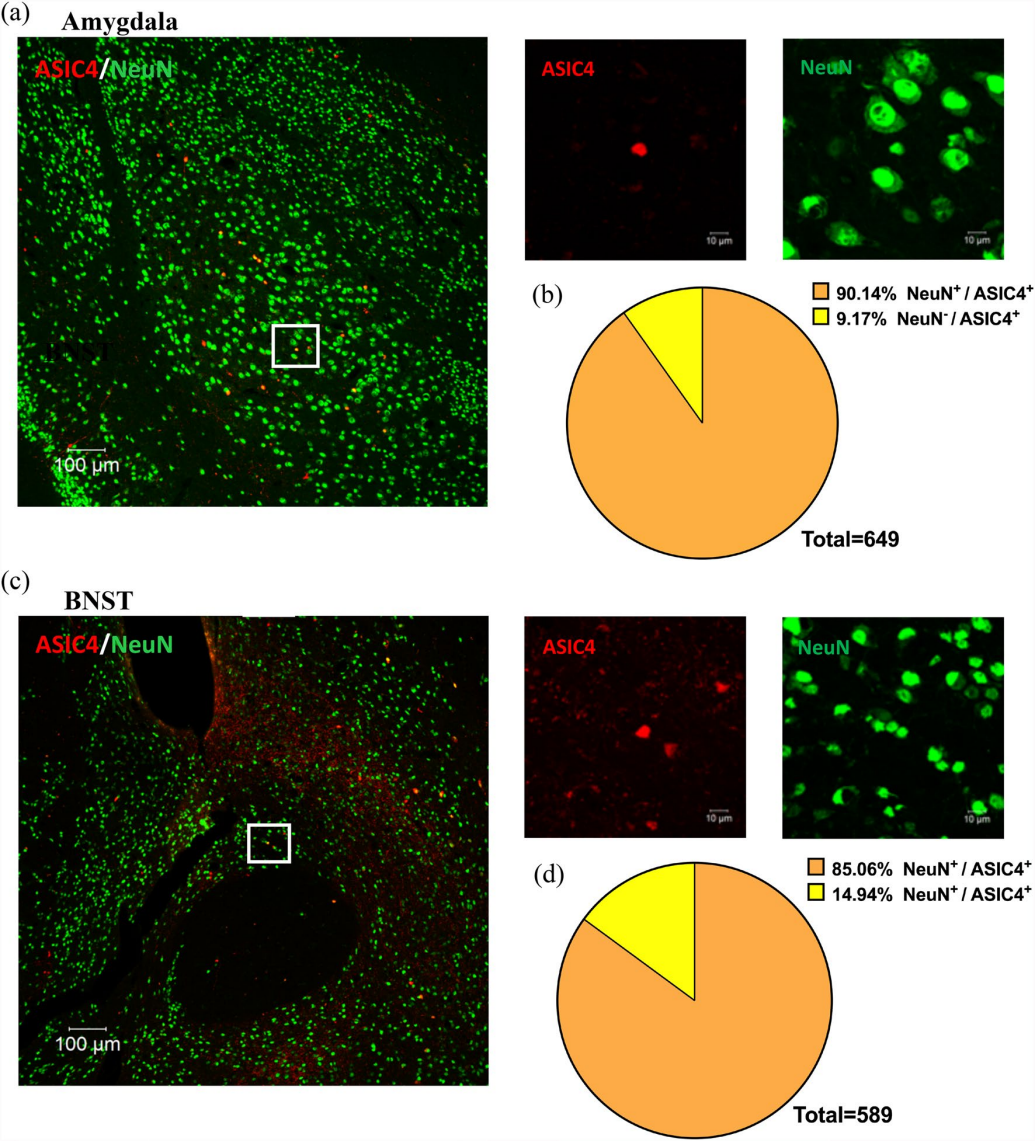
Characterization of ASIC4-expressing cells with neuronal markers. **a** Left panel: representative image of amygdala shows co-localization of NeuN-immunoreactivity with ASIC4-expressing cells in the amygdala of *Asic4*^*CreERT2/*+^*::Td* mice (n = 6). Right panels: enlarged images of the inset in the left panel. **b** Quantification of co-localization of ASIC4 expression and NeuN in the amygdala. **c** Left panel: representative image of amygdala of co-localization of NeuN- immunoreactivity with ASIC4-expressing cells in the BNST of *Asic4*^*CreERT2/*+^*::Td* mice (n = 6). Right panels: enlarged images of the inset in the left panel. **d** Quantification of co-localization of ASIC4 expression and NeuN in the BNST

Last, we used immunohistochemistry to characterize the identity of ASIC4-expressing cells in the amygdala and BNST of *Asic4*^*CreERT2/*+^*::Td* mice. In the amygdala, 90.1% (585/649) of ASIC4-expressing cells were neurons (NeuN-positive) (Fig. 9a, b). In the BNST, 85.1% (501/589) of ASIC4-expressing cells were neurons (Fig. 9c, d).

#### A schematic model of how ASIC4 expression influences ASIC1a activity and anxiety levels.

**Fig. 10:**
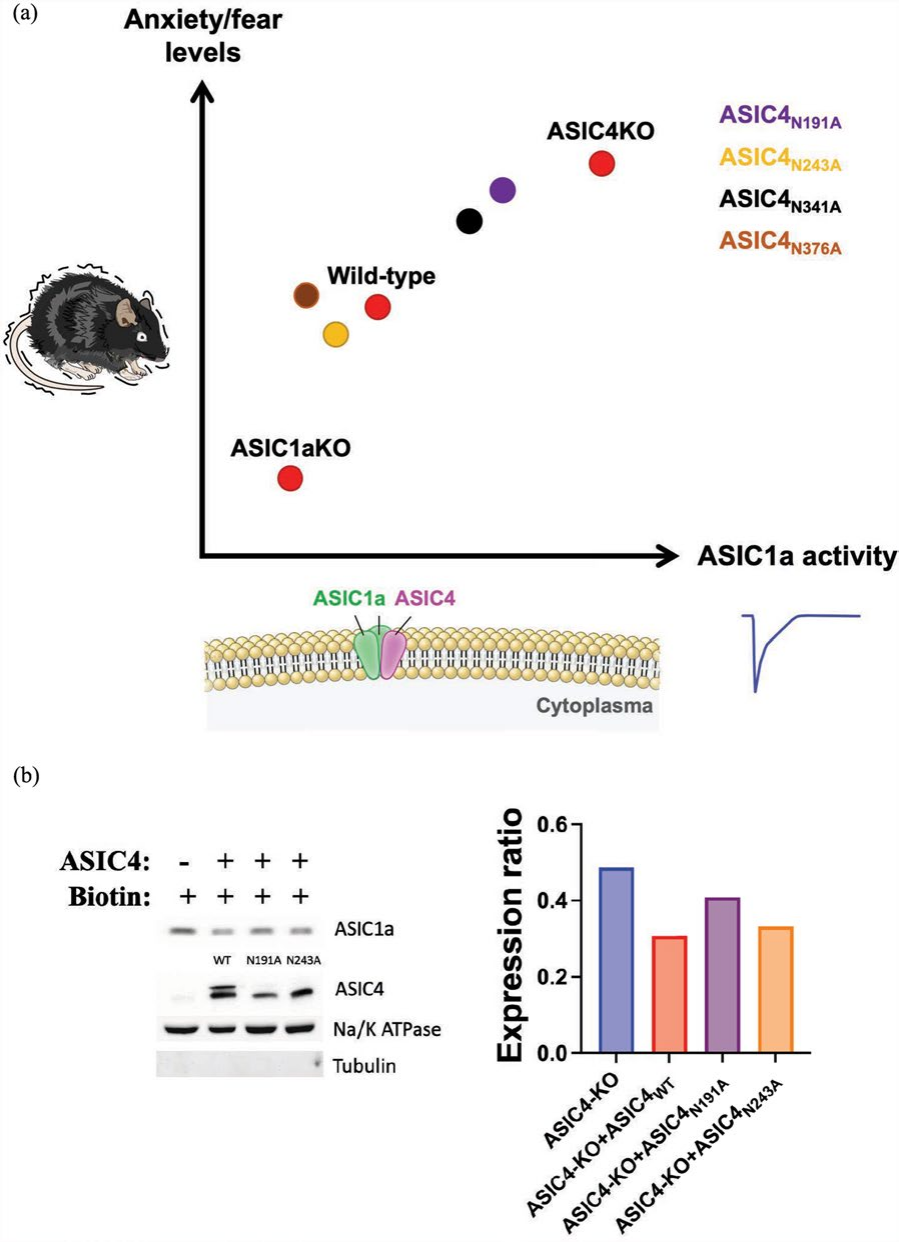
Model illustrates ASIC4's modulation of ASIC1a activity, significantly influencing anxiety levels. **a** This model illustrates a role of ASIC4 as a negative regulator of ASIC1a channel activity, highlighting the critical interaction between ASIC4 and ASIC1a subunits in determining ASIC1a channel function. In Asic4 KO neurons, primarily within the amygdala or BNST, elevated ASIC1a activity is related to increased anxiety/fear levels. Conversely, Asic1a-KO mice exhibit the lowest anxiety/fear levels as compared with wild-type counterparts, which underscores the significance of ASIC1a activity in modulating anxiety and fear responses in these brain regions. **b** Western blot analysis of surface ASIC1a expression in the amygdala in *Asic4*^*−/−*^ mice transfected with AAV-DIO-ASIC4_WT(or Mutant)_ or without transfection. Each protein sample was collected from tissues of 4 mice. Na/K ATPase serves as the loading control, while tubulin is used as a negative control for membrane proteins. Expression ratio of ASIC1a is normalized to Na/K ATPase

This model shows ASIC4 as a negative regulator of ASIC1a channel activity, emphasizing the critical interaction between ASIC4 and ASIC1a subunits in defining ASIC1a function. Elevated ASIC1a activity in ASIC4-KO neurons, mainly in the amygdala or BNST, is linked to increased anxiety/fear levels (Fig. 10a). In contrast, anxiety/fear levels were lower in ASIC1a-KO than wild-type mice, which highlights the role of ASIC1a in modulating these responses. Additionally, mutations at N191 and N341 N-glycosylation sites substantially affected ASIC4's ability to reduce acid-induced *I*_ASIC1a_, amplitude, which heightened anxiety/fear levels.

To know whether the expression of ASIC4 (wild-type or mutants) influences the surface expression of ASIC1a in vivo, we designed a set of experiments to probe the in vivo ASIC1a surface expression in the *Asic4* knockout mice with or without AAV-mediated *Asic4* (wild-type or mutants) rescue in the amygdala (Fig. 10b). Similar to the in vitro results, the in vivo result showed that ASIC1a surface expression was reduced when ASIC4 wild-type was restored in the *Asic4*^*−/−*^ amygdala. ASIC4_N191A_ mutant was less efficient to reduce ASIC1a surface expression as compared with wild-type ASIC4, whereas ASIC4_N243A_ mutant was as potent as ASIC4 wild-type in reducing ASIC1a surface expression. Together, these data provided a proof-of-concept that ASIC4 did reduce ASIC1a surface expression in vivo and the effect was impaired in ASIC4_N191_ mutant.

## Discussion

By using chemo-optogenetic, conditional KO, and AAV-driven gene restoration approaches, we successfully demonstrated that ASIC4-expressing cells of the amygdala and BNST play an essential role in modulating innate anxiety and fear responses in mice. Mechanistically, ASIC4 modulated ASIC1a channel activity (possibly forming heteromeric ASIC1a/ASIC4 channels), which could be affected by N-glycosylation of ASIC4. In brief, the expression of ASIC4 could downregulate ASIC1a channel activity, which is associated with anxiety-like responses (Fig. 10).

In this study, we highlighted ASIC4 as an important regulator to extend our understanding of ASIC1a as a key player in the neurocircuitry of the amygdala and BNST in modulating innate anxiety. We previously showed that ASIC4 KO enhanced but ASIC1a KO reduced anxiety levels in mice [29]. Here we further demonstrated that chemo-optogenetic activation of ASIC4-expressing neurons had a similar effect as conditional ASIC4 KO in the amygdala or BNST in increasing anxiety levels in mice. Therefore, although ASIC4 is widely expressed in the brain, specifically altered ASIC4 expression in the amygdala or BNST may be sufficient for regulating anxiety responses. Of note, ASIC4 was expressed in only 18% and 13% of total cells in the amygdala and BNST, respectively.

About 90% of ASIC4-expressing amygdala/BNST cells also expressed ASIC1a, so the *I*_ASIC1a_ amplitude could be relatively small in ASIC4-expressing cells because ASIC4 would reduce the surface expression of ASIC1a activity and form heteromeric ASIC1a/ASIC4 channels. In contrast, > 50% of amygdala and BNST cells expressed ASIC1a without co-expressing ASIC4. This finding is consistent with our previous functional mapping study showing that ASIC1a is expressed in almost all types of amygdala neurons with a robust acid-induced current; however, a small portion of basolateral amygdala (BLA) fast-spiking interneurons (FSINs) exhibit a small acid-induced current similar to those expressing both ASIC1a and ASIC4 [6]. A major puzzle of these findings is that although ASIC1a/ASIC4 heteromeric channels can contribute only a relatively small acid-induced current, how is the small *I*_ASIC1a_ signal integrated into the amygdala/BNST neuronal response to stress? Further studies should probe how ASIC1a homomeric and ASIC1a/ASIC4 heteromeric channels differentially contribute to neuronal excitability in response to stress and whether the BLA-FSINs are ASIC4-expressing neurons involved in regulating innate anxiety.

A second puzzle of the study is that restoring ASIC4 expression in ASIC4-expressing cells of the amygdala or BNST was sufficient to rescue anxiogenic behaviors of *Asic4*^*−/−*^ mice. Hence, the neurocircuitry of the amygdala and BNST is an integral unit in response to stress. In contrast, both conditional KO and gene restoration in *Asic4*^*−/−*^ studies discounted a role for hippocampal ASIC4 in innate anxiety, although the hippocampus plays an important role in anxiety [32] and fear memory [14, 31], and fear extinction requires ASIC1a-dependent regulation of the ventral hippocampus [36]. A previous study also showed ASIC4 involved in synaptic plasticity in the hippocampus [5]. About 80% of the ASIC4-expressing cells in the amygdala and BNST were neurons in our study. In the amygdala, about 57% of ASIC4-positive neurons are GABAergic, whereas in the BNST, only about 19% of ASIC4-positive neurons are GABAergic (Fig. 9). How these ASIC4-positive neurons work in the amygdala and BNST neural circuitry in response to stress remains for future studies.

We also revealed how glycosylation affects ASIC4 functionality. Among 4 predicted N-glycosylation sites, we identified that mutations on N191 and N341 sites could largely influence the ASIC4 effect on reducing acid-induced *I*_ASIC1a_ amplitude in CHO cells. Although ASIC1a/ASIC4_N191_ and ASIC1a/ASIC4_N341_ still showed smaller *I*_ASIC1a_ amplitude than homomeric ASIC1a, ASIC4_N191_ and ASIC4_N341_ did not rescue the anxiogenic phenotypes of *Asic4*^*−/−*^ mice as did ASIC4_WT_ when expressed in the amygdala or BNST via AAV injection. These data further highlight the importance of maintaining a small *I*_ASIC1a_ amplitude in ASIC4-expresssing cells of the amygdala and BNST for normal innate anxiety responses.

Increasing evidence has shown protons as a neurotransmitter. Additionally, ASIC1a is believed to be the molecular determinant involved in proton-induced postsynaptic currents [13, 17, 18], and its expression is related to long-term synaptic plasticity in the amygdala network [6]. Thus, ASIC1a/ASIC4 heteromeric channels might play an essential role in synaptic transmission and plasticity in the amygdala and BNST apart from sensing acidosis due to CO_2_ inhalation. Although our understanding of “proton-ergic transmission” is still limited and focuses only on ASIC1a homomeric channels, further studies should consider ASIC4 as an important partner in the context of proton-ergic transmission and synaptic plasticity.

Together, our study contributes significantly to understanding the neural mechanisms underlying the ASIC1a-dependent regulation of anxiety and fear. The discovery that ASIC4 co-localizes with ASIC1a and affects ASIC1a-mediated currents deepens our understanding of ion channel interactions and their functional implications in the brain. Understanding the functionality of ASIC1a/ASIC4 heteromeric channels in the amygdala and BNST would bring new insights for the development of new therapeutic strategies for treating anxiety disorders, improving the quality of life for those affected by these conditions.

## Conclusions

This research highlights the significant role of ASIC4 in managing anxiety-related behaviors, emphasizing its interaction with ASIC1a within key brain regions linked to fear and anxiety. By employing advanced chemo-optogenetic and genetic tools, we uncover that activating ASIC4-positive cells enhances anxiety responses, while its absence in specific brain areas leads to anxiety traits. Notably, the targeted removal of ASIC1a from these ASIC4-expressing cells reduces such behaviors, illustrating a counteractive modulation between these channels. Further, we discover crucial glycosylation sites on ASIC4 that affect ASIC1a's surface expression and functionality, influencing anxiety phenotypes. These findings present ASIC4 as a promising target for therapeutic strategies against anxiety, providing foundational knowledge that could lead to the development of novel anxiety treatments.

## Data Availability

Materials related to this study can be obtained from the corresponding author with a reasonable request.

## Abbreviations

ASICs: Acid-sensing ion channels
BNST: Bed nucleus of the stria terminalis
SPASIC: Spinal cord ASIC
AAV: Adeno-associated virus
LMO3: Luminopsin 3
hSyn: Human synapsin promoter
CTZ: Coelenterazine
OF: Open field
EPM: Elevated-plus maze
TMT: 2,4,5-Trimethylthiazoline
EYFP: Enhanced yellow fluorescent protein
ITRs: Inverted terminal repeats
TBS: Tris buffer saline
PFA: Paraformaldehyde
OCT: Optimal cutting temperature
ERK: Extracellular signal-regulated kinase
i.p.: Intraperitoneal
CHO: Chinese hamster ovary
BLA: Basolateral amygdala
FSINs: Fast-spiking interneurons

## References

[1] Ahrens S, Wu MV, Furlan A, Hwang GR, Paik R, Li H, Penzo MA, Tollkuhn J, Li B. A central extended amygdala circuit that modulates anxiety. J Neurosci. 2018;38(24):5567–83. 29844022 10.1523/JNEUROSCI.0705-18.2018 PMC6001032

[2] Akopian AN, Chen CC, Ding Y, Cesare P, Wood JN. A new member of the acid-sensing ion channel family. NeuroReport. 2000;11(10):2217–22. 10923674 10.1097/00001756-200007140-00031

[3] Berglund K, Clissold K, Li HE, Wen L, Park SY, Gleixner J, Klein ME, Lu D, Barter JW, Rossi MA, Augustine GJ, Yin HH, Hochgeschwender U. Luminopsins integrate opto- and chemogenetics by using physical and biological light sources for opsin activation. Proc Natl Acad Sci USA. 2016;113(3):E358-367. 26733686 10.1073/pnas.1510899113 PMC4725499

[4] Calhoon GG, Tye KM. Resolving the neural circuits of anxiety. Nat Neurosci. 2015;18(10):1394–404. 26404714 10.1038/nn.4101 PMC7575249

[5] Chen YJ, Deng SM, Chen HW, Tsao CH, Chen WT, Cheng SJ, Huang HS, Tan BC, Matzuk MM, Flint J, Huang GJ. Follistatin mediates learning and synaptic plasticity via regulation of Asic4 expression in the hippocampus. Proc Natl Acad Sci U S A. 2021;118(39):e2109040118. 34544873 10.1073/pnas.2109040118 PMC8488609

[6] Chiang P-H, Chien T-C, Chen C-C, Yanagawa Y, Lien C-C. ASIC-dependent LTP at multiple glutamatergic synapses in amygdala network is required for fear memory. Sci Rep. 2015;5(1):10143. 25988357 10.1038/srep10143 PMC4437300

[7] Coryell MW, Ziemann AE, Westmoreland PJ, Haenfler JM, Kurjakovic Z, Zha XM, Price M, Schnizler MK, Wemmie JA. Targeting ASIC1a reduces innate fear and alters neuronal activity in the fear circuit. Biol Psychiatry. 2007;62(10):1140–8. 17662962 10.1016/j.biopsych.2007.05.008

[8] Cristofori-Armstrong B, Rash LD. Acid-sensing ion channel (ASIC) structure and function: Insights from spider, snake and sea anemone venoms. Neuropharmacology. 2017;127:173–84. 28457973 10.1016/j.neuropharm.2017.04.042

[9] Davis M, Whalen PJ. The amygdala: vigilance and emotion. Mol Psychiatry. 2001;6(1):13–34. 11244481 10.1038/sj.mp.4000812

[10] Deval E, Gasull X, Noël J, Salinas M, Baron A, Diochot S, Lingueglia E. Acid-sensing ion channels (ASICs): pharmacology and implication in pain. Pharmacol Ther. 2010;128(3):549–58. 20807551 10.1016/j.pharmthera.2010.08.006

[11] Donier E, Rugiero F, Jacob C, Wood JN. Regulation of ASIC activity by ASIC4–new insights into ASIC channel function revealed by a yeast two-hybrid assay. Eur J Neurosci. 2008;28(1):74–86. 18662336 10.1111/j.1460-9568.2008.06282.x

[12] Du J, Reznikov LR, Price MP, Zha XM, Lu Y, Moninger TO, Wemmie JA, Welsh MJ. Protons are a neurotransmitter that regulates synaptic plasticity in the lateral amygdala. Proc Natl Acad Sci USA. 2014;111(24):8961–6. 24889629 10.1073/pnas.1407018111 PMC4066526

[13] Du J, Reznikov LR, Welsh MJ. Expression and activity of acid-sensing ion channels in the mouse anterior pituitary. PLoS ONE. 2014;9(12): e115310. 25506946 10.1371/journal.pone.0115310 PMC4266673

[14] Engin E, Smith KS, Gao Y, Nagy D, Foster RA, Tsvetkov E, Keist R, Crestani F, Fritschy JM, Bolshakov VY, Hajos M, Heldt SA, Rudolph U. Modulation of anxiety and fear via distinct intrahippocampal circuits. Elife. 2016;5: e14120. 26971710 10.7554/eLife.14120 PMC4816644

[15] Faraci FM, Taugher RJ, Lynch C, Fan R, Gupta S, Wemmie JA. Acid-sensing ion channels: novel mediators of cerebral vascular responses. Circ Res. 2019;125(10):907–20. 31451088 10.1161/CIRCRESAHA.119.315024 PMC6813889

[16] Felix-Ortiz AC, Beyeler A, Seo C, Leppla CA, Wildes CP, Tye KM. BLA to vHPC inputs modulate anxiety-related behaviors. Neuron. 2013;79(4):658–64. 23972595 10.1016/j.neuron.2013.06.016 PMC4205569

[17] González-Inchauspe C, Gobetto MN, Uchitel OD. Modulation of acid sensing ion channel dependent protonergic neurotransmission at the mouse calyx of Held. Neuroscience. 2020;439:195–210. 31022462 10.1016/j.neuroscience.2019.04.023

[18] González-Inchauspe C, Urbano FJ, Di Guilmi MN, Uchitel OD. Acid-sensing ion channels activated by evoked released protons modulate synaptic transmission at the mouse calyx of held synapse. J Neurosci. 2017;37(10):2589–99. 28159907 10.1523/JNEUROSCI.2566-16.2017 PMC6596635

[19] Graeff FG, Silveira MC, Nogueira RL, Audi EA, Oliveira RM. Role of the amygdala and periaqueductal gray in anxiety and panic. Behav Brain Res. 1993;58(1–2):123–31. 8136040 10.1016/0166-4328(93)90097-a

[20] Gründer S, Geissler HS, Bässler EL, Ruppersberg JP. A new member of acid-sensing ion channels from pituitary gland. NeuroReport. 2000;11(8):1607–11. 10852210 10.1097/00001756-200006050-00003

[21] Gründer S, Pusch M. Biophysical properties of acid-sensing ion channels (ASICs). Neuropharmacology. 2015;94:9–18. 25585135 10.1016/j.neuropharm.2014.12.016

[22] Hung C-H, Chin Y, Fong Y-O, Lee C-H, Han D-S, Lin J-H, Sun W-H, Chen C-C. Acidosis-related pain and its receptors as targets for chronic pain. Pharmacol Ther. 2023;247: 108444. 37210007 10.1016/j.pharmthera.2023.108444

[23] Janak PH, Tye KM. From circuits to behaviour in the amygdala. Nature. 2015;517(7534):284–92. 25592533 10.1038/nature14188 PMC4565157

[24] Jasti J, Furukawa H, Gonzales EB, Gouaux E. Structure of acid-sensing ion channel 1 at 1.9 A resolution and low pH. Nature. 2007;449(7160):316–23. 17882215 10.1038/nature06163

[25] Jing L, Chu XP, Jiang YQ, Collier DM, Wang B, Jiang Q, Snyder PM, Zha XM. N-glycosylation of acid-sensing ion channel 1a regulates its trafficking and acidosis-induced spine remodeling. J Neurosci. 2012;32(12):4080–91. 22442073 10.1523/JNEUROSCI.5021-11.2012 PMC3322463

[26] Kreple CJ, Lu Y, Taugher RJ, Schwager-Gutman AL, Du J, Stump M, Wang Y, Ghobbeh A, Fan R, Cosme CV, Sowers LP, Welsh MJ, Radley JJ, LaLumiere RT, Wemmie JA. Acid-sensing ion channels contribute to synaptic transmission and inhibit cocaine-evoked plasticity. Nat Neurosci. 2014;17(8):1083–91. 24952644 10.1038/nn.3750 PMC4115047

[27] Lim J, Tai H-H, Liao W-H, Chu Y-C, Hao C-M, Huang Y-C, Lee C-H, Lin S-S, Hsu S, Chien Y-C, Lai D-M, Chen W-S, Chen C-C, Wang J-L. ASIC1a is required for neuronal activation via low-intensity ultrasound stimulation in mouse brain. Elife. 2021;10:e61660. 34569932 10.7554/eLife.61660 PMC8510583

[28] Lin SH, Cheng YR, Banks RW, Min MY, Bewick GS, Chen CC. Evidence for the involvement of ASIC3 in sensory mechanotransduction in proprioceptors. Nat Commun. 2016;7:11460. 27161260 10.1038/ncomms11460 PMC4866049

[29] Lin SH, Chien YC, Chiang WW, Liu YZ, Lien CC, Chen CC. Genetic mapping of ASIC4 and contrasting phenotype to ASIC1a in modulating innate fear and anxiety. Eur J Neurosci. 2015;41(12):1553–68. 25828470 10.1111/ejn.12905

[30] Lin SH, Sun WH, Chen CC. Genetic exploration of the role of acid-sensing ion channels. Neuropharmacology. 2015;94:99–118. 25582292 10.1016/j.neuropharm.2014.12.011

[31] Liu X, Ramirez S, Pang PT, Puryear CB, Govindarajan A, Deisseroth K, Tonegawa S. Optogenetic stimulation of a hippocampal engram activates fear memory recall. Nature. 2012;484(7394):381–5. 22441246 10.1038/nature11028 PMC3331914

[32] Shi HJ, Wang S, Wang XP, Zhang RX, Zhu LJ. Hippocampus: molecular, cellular, and circuit features in anxiety. Neurosci Bull. 2023;39(6):1009–26. 36680709 10.1007/s12264-023-01020-1 PMC10264315

[33] Taugher RJ, Lu Y, Wang Y, Kreple CJ, Ghobbeh A, Fan R, Sowers LP, Wemmie JA. The bed nucleus of the stria terminalis is critical for anxiety-related behavior evoked by CO_2_ and acidosis. J Neurosci. 2014;34(31):10247–55. 25080586 10.1523/JNEUROSCI.1680-14.2014 PMC4115136

[34] Verkest C, Salinas M, Diochot S, Deval E, Lingueglia E, Baron A. Mechanisms of action of the peptide toxins targeting human and rodent acid-sensing ion channels and relevance to their in vivo analgesic effects. Toxins. 2022;14(10):709. 36287977 10.3390/toxins14100709 PMC9612379

[35] Waldmann R, Champigny G, Bassilana F, Heurteaux C, Lazdunski M. A proton-gated cation channel involved in acid-sensing. Nature. 1997;386(6621):173–7. 9062189 10.1038/386173a0

[36] Wang Q, Wang Q, Song XL, Jiang Q, Wu YJ, Li Y, Yuan TF, Zhang S, Xu NJ, Zhu MX, Li WG, Xu TL. Fear extinction requires ASIC1a-dependent regulation of hippocampal-prefrontal correlates. Sci Adv. 2018;4(10):eaau3075. 30417090 10.1126/sciadv.aau3075 PMC6223961

[37] Wemmie JA, Askwith CC, Lamani E, Cassell MD, Freeman JH Jr, Welsh MJ. Acid-sensing ion channel 1 is localized in brain regions with high synaptic density and contributes to fear conditioning. J Neurosci. 2003;23(13):5496–502. 12843249 10.1523/JNEUROSCI.23-13-05496.2003 PMC6741257

[38] Wemmie JA, Taugher RJ, Kreple CJ. Acid-sensing ion channels in pain and disease. Nat Rev Neurosci. 2013;14(7):461–71. 23783197 10.1038/nrn3529 PMC4307015

[39] Weng JY, Lin YC, Lien CC. Cell type-specific expression of acid-sensing ion channels in hippocampal interneurons. J Neurosci. 2010;30(19):6548–58. 20463218 10.1523/JNEUROSCI.0582-10.2010 PMC6632567

[40] Wu PY, Huang YY, Chen CC, Hsu TT, Lin YC, Weng JY, Chien TC, Cheng IH, Lien CC. Acid-sensing ion channel-1a is not required for normal hippocampal LTP and spatial memory. J Neurosci. 2013;33(5):1828–32. 23365222 10.1523/JNEUROSCI.4132-12.2013 PMC6619135

[41] Xiong ZG, Zhu XM, Chu XP, Minami M, Hey J, Wei WL, MacDonald JF, Wemmie JA, Price MP, Welsh MJ, Simon RP. Neuroprotection in ischemia: blocking calcium-permeable acid-sensing ion channels. Cell. 2004;118(6):687–98. 15369669 10.1016/j.cell.2004.08.026

[42] Ziemann AE, Allen JE, Dahdaleh NS, Drebot II, Coryell MW, Wunsch AM, Lynch CM, Faraci FM, Howard MA, Welsh MJ, Wemmie JA. The amygdala is a chemosensor that detects carbon dioxide and acidosis to elicit fear behavior. Cell. 2009;139(5):1012–21. 19945383 10.1016/j.cell.2009.10.029 PMC2808123

